# Surface Depression and Wetland Water Storage Improves Major River Basin Hydrologic Predictions

**DOI:** 10.1029/2019WR026561

**Published:** 2020-07-06

**Authors:** Adnan Rajib, Heather E. Golden, Charles R. Lane, Qiusheng Wu

**Affiliations:** 1 Department of Environmental Engineering, Texas A&M University, Kingsville, TX, USA; 2 Formerly at Oak Ridge Institute for Science and Education, US Environmental Protection Agency, Office of Research and Development, Cincinnati, OH, USA; 3 US Environmental Protection Agency, Office of Research and Development, Cincinnati, OH, USA; 4 Department of Geography, University of Tennessee, Knoxville, TN, USA

## Abstract

Surface water storage in small yet abundant landscape depressions—including wetlands and other small waterbodies—is largely disregarded in conventional hydrologic modeling practices. No quantitative evidence exists of how their exclusion may lead to potentially inaccurate model projections and understanding of hydrologic dynamics across the world’s major river basins. To fill this knowledge gap, we developed the first-ever major river basin-scale modeling approach integrating surface depressions and focusing on the 450,000-km^2^ Upper Mississippi River Basin (UMRB) in the United States. We applied a novel topography-based algorithm to estimate areas and volumes of ~455,000 surface depressions (>1 ha) across the UMRB (in addition to lakes and reservoirs) and subsequently aggregated their effects per subbasin. Compared to a “no depression” conventional model, our depression-integrated model (a) improved streamflow simulation accuracy with increasing upstream abundance of depression storage, (b) significantly altered the spatial patterns and magnitudes of water yields across 315,000 km^2^ (70%) of the basin area, and (c) provided realistic spatial distributions of rootzone wetness conditions corresponding to satellite-based data. Results further suggest that storage capacity (i.e., volume) alone does not fully explain depressions’ cumulative effects on landscape hydrologic responses. Local (i.e., subbasin level) climatic and geophysical drivers and downstream flowpath-regulating structures (e.g., reservoirs and dams) influence the extent to which depression storage volume in a subbasin causes hydrologic effects. With these new insights, our study supports the integration of surface depression storage and thereby catalyzes a reassessment of current hydrological modeling and management practices for basin-scale studies.

## 1. Introduction

With the evolution of powerful computational resources and globally available geospatial data sets (e.g., weather and topography; Bauer et al., 2015; Gasper et al., 2014; Yang et al., 2018), current-generation hydrologic models are increasingly being used across large spatial extents. Consequently, an abundance of process-based models has been developed to predict current and potential future states of water availability, water hazards, and human-water interactions across the world’s major river basins (e.g., Du et al., 2018; Maxwell et al., 2015; Pokhrel et al., 2018; Prudhomme et al., 2014; Salas et al., 2018; Schewe et al., 2014; Veldkamp et al., 2017; Wood et al., 2011).

Though state-of-the-science basin-scale models encapsulate hydrologic dynamics by effectively curve-fitting observed data (Clark et al., 2017; Fatichi et al., 2016), it is commonly accepted that current major river basin-scale (hereafter referred to as basin scale) models lack the specificity and fidelity required to adequately represent system patterns and emergent processes. Today’s models may therefore be providing the right answers for the wrong reasons (Kirchner, 2006; Ruddell et al., 2019). This problem persists due to limited process representations within hydrologic models along with input data uncertainty and parameter equifinality (Beven & Cloke, 2012; Kauffeldt et al., 2013). Accordingly, researchers are tackling these challenges through improved soil moisture accounting, surface-subsurface interactions, vegetation dynamics, and river routing mechanisms, coupled with spatially distributed assimilation/calibration schemes using remotely sensed data (e.g., Camporese et al., 2015; Endrizzi et al., 2014; Kollet & Maxwell, 2008; Rajib, Merwade, & Yu, 2018; Shen & Phanikumar, 2010). However, there has been surprisingly little emphasis on the hydrologic influence of surface depressions and how including their storage capacities could further enhance models’ physical realism and prediction accuracy across the world’s major river basins.

Surface depressions on the landscape vary greatly in size, ranging from relatively small, unmanaged water storage systems to large, regularly managed waterbodies such as lakes and reservoirs. Small surface depressions include wetlands embedded within uplands or those along river corridors, ponds, and other similar small waterbodies (Biggs et al., 2017). Water storage is a primary function of these small aquatic systems, yet lakes and reservoirs are frequently the primary focus when quantifying water availability because of their perceived large water storage capacities. Small surface depressions (hereafter referred to as surface depressions) are therefore traditionally not integrated into estimates and models of basin-scale hydrologic dynamics (Dang et al., 2020; Gao et al., 2012; Liu et al., 2018; Zhou et al., 2016). Surface depressions are largely overlooked in basin-scale hydrologic model applications, despite being abundant landscape features (Downing, 2010). Yet their potential influence on watershed hydrology has recently become well recognized (e.g., Alexander et al., 2018; Cohen et al., 2016; Creed et al., 2017; Golden et al., 2017; Lane et al., 2018; Yu & Harbor, 2019).

Surface depressions throughout watersheds perform important sink-lag-source functions affecting downstream waters (Brooks et al., 2018; Gleason et al., 2007; Lane et al., 2018; Leibowitz et al., 2008; Shook & Pomeroy, 2011; USEPA, 2015; Wilcox et al., 2011). An individual depression receives water from an upstream area and may store this water for long periods (sink), allow temporary retention and a delayed flux release (lag), or cycle through evapotranspiration, seepage, overland, and lateral outflow processes to produce a net outgoing flux that reaches other waterbodies (source) (McLaughlin et al., 2014; Rains et al., 2016; Rains, 2011). The presence of numerous surface depressions can therefore considerably affect the cumulative hydrologic response of a watershed to different drivers (changes in climate and land management), collectively impacting the magnitude, timing, and spatial pattern of water flow and distribution (Chu, 2017; Golden et al., 2016; Huang et al., 2011; Jones et al., 2019; Vanderhoof et al., 2016).

With the improved process-level understanding of how surface depressions affect watershed hydrology and increased opportunities for quantifying their water storage capacities through recent geospatial data developments (Jan et al., 2018; Jones et al., 2019), the interdisciplinary hydro-geoscience community has recently started including surface depression storage in spatially explicit models with varying levels of spatial heterogeneity, physical interactions, and connectivity (e.g., Ameli & Creed, 2017; Blanchette et al., 2019; Evenson, Golden, et al., 2018; Evenson, Jones, et al., 2018; Fossey & Rousseau, 2016; Golden et al., 2019; Nasab & Chu, 2020). However, these model applications have been limited to small or meso-watershed scales, with drainage areas ranging from a few hectares to several thousand square kilometers. A depression-integrated basin-scale model does not exist in the literature nor does evidence of how these depressional storage systems affect basin-scale hydrologic dynamics.

Despite ubiquitous scientific evidence and the emerging community consensus on the hydrological, biophysical, and biogeochemical importance of surface depressions, calls for future directions in water predictions (Bierkens et al., 2015; Clark et al., 2015, 2017; Fatichi et al., 2016) have not explicitly acknowledged the potentially substantive effects of integrating surface depression storage in basin-scale models. Disregarding surface depression storage thus remains a persistent issue in hydro-geoscience (Golden et al., 2014, 2019). While the effects of depression storage on model results depend on depressions’ abundance and spatial distribution over the landscape (Ameli & Creed, 2019; Evenson, Golden, et al., 2018), the widespread practice of disregarding these natural landscape features elicits a serious concern: What if our model-based understanding of basin-scale hydrologic responses to future environmental conditions are potentially flawed? We submit that because of the omission of surface depressions, management and sustainability decisions based on the existing basin-scale hydrologic models may be imprecise at best and ineffective at worst.

The objective of our study is to quantitatively assess whether the inclusion of surface depressions in a process-based model improves hydrologic simulations across the world’s major river basins compared to the conventional approach of disregarding them. We conduct our analyses by developing the first-ever depression-integrated basin-scale modeling approach. We focus on the 450,000-km^2^ Upper Mississippi River Basin (UMRB) in the United States as a test case and ask the following questions: (a) How do depression-integrated model simulations respond to an increased upstream abundance of surface depressions? (b) Do surface depressions with small storage capacities substantially alter subbasin hydrologic responses? (c) Do changes imposed by integrating surface depressions into a basin-scale watershed model suggest enhanced physical realism in simulated water balances? We address these questions by (a) focusing on streamflow accuracy at select sites across the URMB, (b) assessing variation in subbasin water yields, and (c) evaluating the spatial consistency of subbasin rootzone wetness conditions with satellite-based estimates. Answers to our study’s questions could pave the way for a new surface depression-integrated hydrologic modeling paradigm and provide important insights for those across the globe studying, managing, and modeling surface water resources.

## 2. Methodology

We developed two contrasting model configurations using the Soil and Water Assessment Tool (SWAT; Neitsch et al., 2011) to determine how surface depressions alter hydrologic dynamics across the UMRB:

1. *The conventional model*: Surface depressions were not explicitly included in the model, an approach traditionally followed in hydrologic modeling practices.
2. *The depression-integrated model*: The same setup as in the conventional model, except surface depressions and their associated water storage capacities, were explicitly included.

In the following subsections, we outline the rationale for selecting UMRB as our study area, methods used to set up the UMRB model, descriptions of model configurations (with and without surface depressions), and the calibration-verification procedure.

### 2.1. Study Area: The Upper Mississippi River Basin

**Figure 1.**
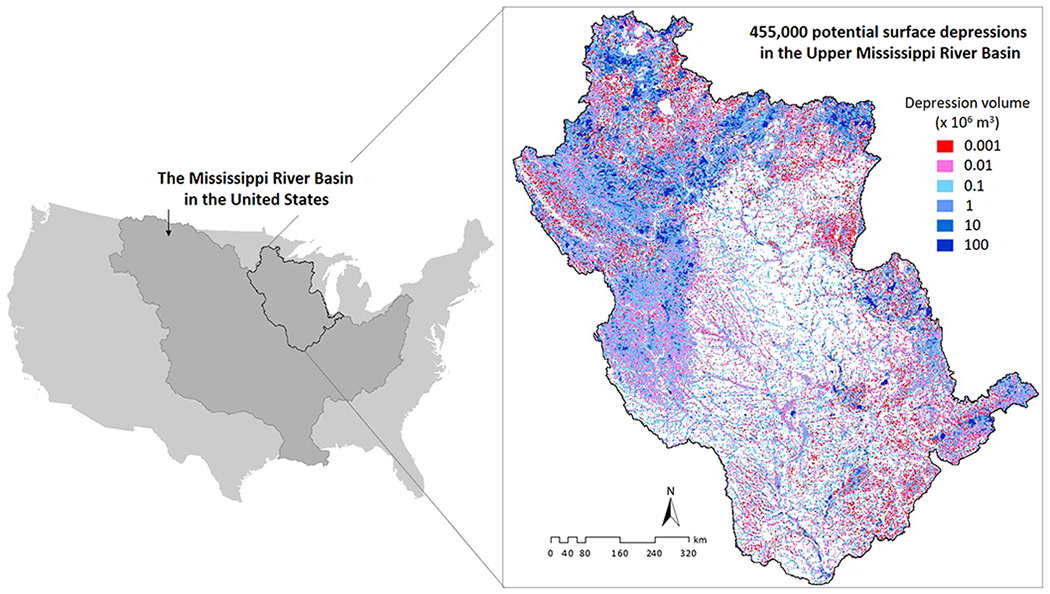
Potential surface depressions across the ~450,000-km^2^ Upper Mississippi River Basin (UMRB) in the United States. Approximately 455,000 individual depressions were identified from the 30-m resolution topographic data.

The 450,000-km^2^ UMRB (Figure 1) is predominantly an agricultural landscape, responsible for more than 50% of the total corn and soybean production in the United States (Schnitkey, 2013). It drains 15% of the Mississippi River Basin, which covers over 40% of the contiguous land area of the United States. The UMRB also contributes nearly half of the average annual nitrogenous pollution to the Gulf of Mexico, exacerbating seasonal hypoxic conditions (Goolsby et al., 2000; Turner et al., 2008). Like other major basins across the world (e.g., the Mekong in Southeast Asia; Pokhrel et al., 2018), potential changes in UMRB’s hydrologic dynamics from natural and anthropogenic influences could limit fresh water availability and agricultural production, provoke flood and drought hazards, and intensify water quality problems (Quinn et al., 2019; Rajib & Merwade, 2017; Secchi et al., 2011; Wing et al., 2018). Therefore, outcomes from using this important heterogeneous region to understand the influence of surface depression storage may have immediate management implications—both to the UMRB and to other major basins worldwide. Furthermore, as a globally significant basin, the UMRB has ample stream gage stations with long-term data availability which makes model setup, evaluation, and hypothesis testing efficient.

### 2.2. The Hydrologic Model

SWAT is a process-based, semi-distributed, continuous-time hydrologic model capable of simulating landscape water balances, water quality, crop yield, and best management practices related to environmental changes (Arnold et al., 2012; Gassman et al., 2007; Neitsch et al., 2011). SWAT divides a watershed into multiple subbasins, which are further discretized into Hydrologic Response Units (HRUs) with identical land use, slope, and soil characteristics (Neitsch et al., 2011). Increased research interest in the hydrological, biophysical, and biogeochemical functions of surface depressions has led to a concomitant trend of depression-integrated SWAT applications in small- or meso-scale watersheds (e.g., Almendinger et al., 2014; Evenson et al., 2016; Evenson et al., 2018,b; Liu et al., 2008; Nasab et al., 2017; Wang et al., 2008). Because SWAT is also frequently used for modeling processes within major river basins worldwide (e.g., Abbaspour et al., 2015; Du et al., 2018; Rajib et al., 2020; Schuol et al., 2008), it is an ideal tool to explore how surface depressions influence basin-scale hydrologic predictions.

Geospatial input data, primarily topography, land use, soil texture, and weather forcing required to set up the URMB model, are summarized in Table 1. In the conventional model configuration, we divided the UMRB into 279 subbasins (with an average subbasin drainage area of 1,613 km^2^). We initially discretized this model into 6,500 HRUs during our preliminary modeling experiment. However, to reduce computational intensity, we tested whether a discretized model using the 279 subbasins alone, that is, without 6,500 HRUs, would be sufficient for our research needs. When we compared the average annual water yield outputs, the 6,500 HRU-scale experimental model did not meaningfully differ from the subbasin-scale model. Therefore, following Du et al. (2018), Faramarzi et al. (2017), and Schuol et al. (2008), we elected to minimize computational overhead by not further discretizing the subbasins into smaller spatial units (e.g., the HRUs).

Subsurface drainage in low-slope, poorly drained agricultural areas is a key factor affecting UMRB’s water balance. Therefore, artificial subsurface tiles drain ~20% of the total basin area (Srinivasan et al., 2010). To explicitly quantify these effects, we delineated the tiled subbasins using a geodatabase which was consistent with our land use and soil inputs (Nakagaki & Wieczorek, 2016; Table 1; Figure S1). We adopted the tile-drain parameter values that are most commonly used in the Midwestern United States (Hutchinson & Christiansen, 2013; Moriasi et al., 2012; see Figure S1).

We used the Penman-Monteith equation for evapotranspiration estimation (Rajib et al., 2018), the Curve Number method for infiltration-surface runoff generation, and the variable storage method for river routing (Neitsch et al., 2011). We parameterized each of the 15 major lakes and reservoirs in the UMRB (Lehner & Doll, 2004) using their unique hydraulic design and storage-discharge information obtained from the U.S. National Inventory of Dams (USACE, 2018; Table 1). Specifically, we incorporated the maximum water surface area and storage volume, area and volume in normal operating conditions, and design release rate at the dam outlet. Although this release rate was used to constrain the model’s reservoir routing scheme, we fit (via direct insertion assimilation) available daily dam outflow data at five locations (supporting information Figure S2).

The simulation length for both model configurations was 10 years (2008–2017). This 10-year simulation allowed us to analyze the role of surface depression storage based on long-term average model outputs while also minimizing the sensitivity of our interpretations to the interannual variations in climatic conditions. The significance of difference between the two output time-series (conventional and depression-integrated simulations) was quantified with a paired *t* test (KSU Libraries, 2017).

### 2.3. Model Configurations

#### 2.3.1. The Conventional “No-Depression” Model

The conventional model described in section 2.2 did not include surface depressions and their storage volume in hydrologic simulations, representing the traditional approach for watershed modeling, hypothesis testing, and management decisions. This configuration was our baseline to measure the degree of change in simulated streamflow, water yield, and rootzone soil moisture when surface depressions were included in the subsequent configuration.

#### 2.3.2. The Depression-Integrated Model

The depression-integrated model was the same as the conventional model, except with the inclusion of surface depressions and their water storage capacities (i.e., maximum fillable volume). Use of elevation (to compute depression depths) and areal extent has emerged as an efficient way to estimate surface-water storage volume (Gao, 2015). Following this trend, we delineated potential surface depressions from topographic data (digital elevation model, DEM) and estimated the maximum surface area and storage volume therein using a novel, efficient algorithm (Wu et al., 2019; Wu & Lane, 2016). Briefly, we first filled the original 30-m DEM using a depression-filling algorithm to create a “depressionless” DEM. Subsequently, we subtracted the original DEM from the filled DEM to identify ~455,000 depressions (each >1 ha), then we estimated the area and volume of every depression based on a statistical analysis of the DEM cells comprising that depression (see Wu & Lane, 2016; Wu et al., 2019 for details). In a previous study (i.e., Wu et al., 2019), we validated the depression volumes in selected subbasins where high-resolution bathymetric LiDAR DEMs are available.

To avoid DEM’s vertical inaccuracy propagating into estimated depression volumes, some studies considered empirical area-to-volume conversion equations (e.g., Gleason et al., 2007; Jones et al., 2017). These empirical approaches may lack accuracy for depressions with complex morphometry (see, e.g., Wu & Lane, 2016). Further, most of the existing global databases (see Aires et al., 2017; Lehner & Doll, 2004; Pekel et al., 2016; Tootchi et al., 2019; Yamazaki et al., 2015) do not provide estimates of depression volumes (i.e., databases that are available are mostly useful for generating estimates of surface areas, though at coarse spatial resolutions). Our approach to efficiently estimate both area and volume of surface depressions from any given topography data (Wu et al., 2019; Wu & Lane, 2016) fills these information gaps and affords a reproducible, first step at quantifying depression-effects in the world’s major river basins.

The individual depression-volume in the UMRB ranged between 5 × 10^−2^m^3^ and 1 × 10^8^ m^3^ with the basin-average volume around 6 × 10^4^ m^3^ (Figure 1). However, we eliminated depressions smaller than 1 ha to keep computational overhead reasonable. Further, to distinguish the cumulative effect of surface depressions from that of the large, regularly managed waterbodies, which are traditionally included in hydrologic modeling, both our model configurations explicitly integrated hydraulic geometry and storage-discharge data for the major lakes and reservoirs (section 2.2). As a result, we excluded those lakes and reservoirs from the DEM-based area-volume estimation. Any depression that fell within the channel width was also removed to avoid overestimating in-channel storage. Finally, we aggregated the depression area and volume for each subbasin to have a parsimonious simulation of depression hydrology across the UMRB (see below).

#### 2.3.3. Conceptualizing Depressional Storage Capacity

The subbasin-level aggregation or “lumping” of surface depressions essentially follows the hydrologic equivalent wetland (HEW) concept (Wang et al., 2008). It is assumed that the depressions within a subbasin exhibit similar hydrologic responses; if all these depressions are virtually aggregated to form a single HEW, their cumulative subbasin-level effect would be equivalent to what the HEW would alone produce. This concept, often implemented in process-based semi-distributed models (Feng et al., 2013; Fossey et al., 2015; Neitsch et al., 2011; Rahman et al., 2016; Voldseth et al., 2007), is associated with empirical power-law equations to simulate the hydrology of surface depressions via time-varying area-volume relationships (see, e.g., Neitsch et al., 2011). The power-law equations in our model included shape factors, defined as a function of existing water storage volume in the HEW, to represent the variable non-wetted depression geometry and hence the potential fillable volume in successive time steps. Because HEWs are aggregated virtual waterbodies without having specific geolocation within respective subbasins, it is infeasible to represent explicit depression-to-stream connectivity and hydrologic transport between a depression and its corresponding upland drainage area (see, e.g., SWAT-based algorithms developed by Evenson et al., 2016; Evenson, Golden, et al., 2018; Lee et al., 2018; Nasab et al., 2017). Therefore, we estimated inflow from the entire non-depressional portion of the subbasin at every simulation time step. According to this inflow, and the evapotranspiration and seepage losses that were extracted from the existing storage volume, we estimated the “net change” of water storage in a HEW (note, storage capacity = maximum fillable volume; section 2.3.2). Finally, this “net change” was either retained in the HEW or directly poured in the channel, using the maximum storage volume of the HEW as the threshold prior to it “spilling” (Neitsch et al., 2011). Despite such parsimony, HEW conceptualization produces acceptable results in diverse geophysical settings (e.g., Feng et al., 2013; Rahman et al., 2016).

The depression-integrated UMRB model required defining two additional parameters specific to surface depression hydrology (hydraulic conductivity and rate of evaporation). Suitable values for these two parameters were adopted from Golden et al. (2019) and kept spatially constant throughout the basin (see Table S1).

### 2.4. Calibration and Verification

**Figure 2.**
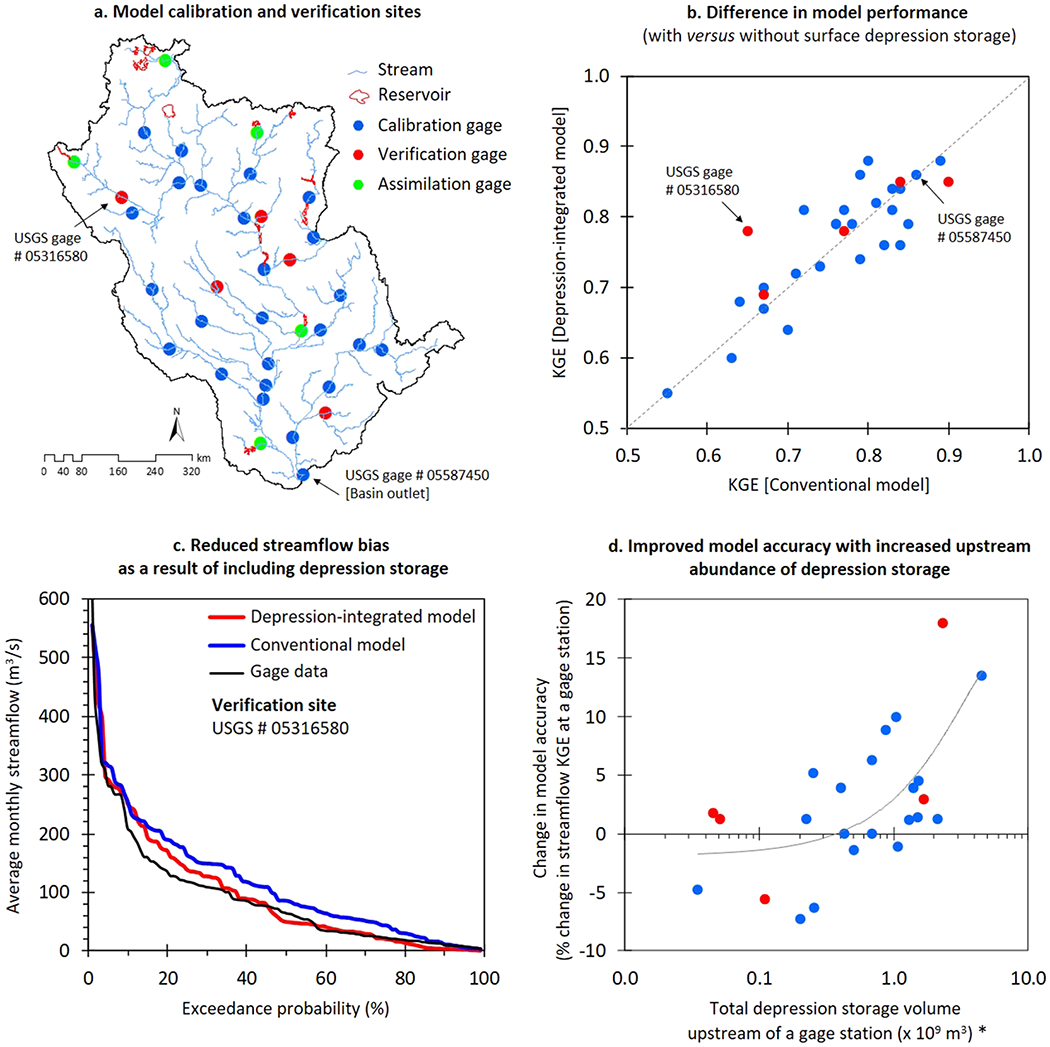
(a) Streamflow gage stations (*N* = 30) for model calibration and verification (see Figure S3 for corresponding gage station IDs). Major lakes and reservoirs included in the model (*N* = 15) and the corresponding dam outflow data assimilation locations (*N* = 5) are also shown here; (b) change in model performance between conventional and depression-integrated configurations (in terms of Kling-Gupta efficiency, KGE); (c) evidence of reduced bias in simulated streamflow due to the inclusion of surface depression storage; (d) relative improvement in model accuracy at gage stations with increasing upstream abundance of depression storage. Here, asterisk in the *x*-axis indicates elimination of six gage stations from the main channel of the river network to avoid spatial autocorrelation.

We followed an identical calibration protocol for both model configurations (conventional and depression integrated). The calibration involved average monthly streamflow data from 25 U.S. Geological Survey gage stations across the basin (Figures 2a and S3). The calibration length was 10 years (2008–2017) following a 3-year initialization (2005–2007). We applied a multisite optimization scheme using the Sequential Uncertainty Fitting algorithm-version 2 (SUFI-2), which is a semi-automated inverse modeling procedure available inside SWAT-CUP platform (Abbaspour, 2015). For the conventional model configuration, calibration parameters and their initial ranges (to start the SUFI-2 optimization process) were adopted from a previous UMRB SWAT setup (Rajib & Merwade, 2017; see Table S1). For consistency, the depression-integrated model used an identical set of calibration parameters. A weighted Kling-Gupta efficiency (KGE; Gupta et al., 2009; Knoben et al., 2019; Rajib, Evenson, et al., 2018) was the objective function to measure the association between simulated and gage streamflow data. KGE ranges from −∞ to 1. Model accuracy increases as KGE values move closer to 1. SUFI-2 identified the best parameter combination corresponding to the highest possible KGE values and hence the most optimal streamflow simulations across all calibration locations.

To enable a post hoc evaluation that is independent of calibration, we verified the most optimal streamflow for the same simulation period (2008–2017) at five separate gage stations not included in the calibration process (Figure 2a and S3). The verification sites represent different drainage areas and locations within the basin, and more importantly, largely different populations of surface depressions in their respective upstream areas. Further, we checked the spatial consistency of the models’ soil moisture accounting with satellite-based estimates (Soil Moisture Active Passive [SMAP] mission; Table 1). Briefly, we took the seasonal-average rootzone SMAP data (volumetric water content) for the 2016 crop growing season (June–August), aggregated estimates at subbasin level, and then performed a spatial normalization across all subbasins to create a dimensionless relative wetness index. This spatial aggregation-normalization approach reduced the effects of uncertainties common to any remotely sensed data (e.g., Chen et al., 2019). It also minimized the conceptual and structural inconsistencies between SMAP and a hydrologic model (e.g., spatial and vertical heterogeneity, and rootzone structure; Koster et al., 2018), therefore affording their one-to-one comparison at a target spatial scale (here, subbasins). To facilitate a focused assessment of depression-effects, we compared SMAP data with model simulations over a 18,300-km^2^ area at the depression-dominated north-west region of the basin (see Figure 1).

## 3. Results

### 3.1. Effect of Surface Depression Storage on Streamflow Accuracy

The inclusion of surface depression storage affected streamflow simulation accuracy with higher or unchanged KGE values compared to the conventional model at 67% of the target stream gage locations (increased in 16, unchanged in 4, and decreased in 10; *N* = 30; Figures 2a and 2b). Because the primary difference in the two models was the absence or presence of surface depressions, our results suggest that the lack of surface depression storage was the driving factor for the conventional model to largely overestimate streamflow in areas where depression storage volumes are substantive (e.g., verification site #05316580 in Figure 2c). The relative improvements in streamflow KGEs at the target locations were correlated with their respective upstream abundance of surface depression storage (Figure 2d)—a prominent signature of enhanced process representation in the depression-integrated model. In fact, at stream gages where KGE decreased slightly, upgradient surface depression storage was minimal (Figure 2d). However, while model improvements may be reflected in KGE values closer to 1 for simulated streamflow, other improvements associated with making the model more physically realistic via increased inclusivity of hydrologic processes associated with surface depressions (e.g., spatially varying changes in water yields) cannot be fully captured by this objective function.

**Figure 3.**
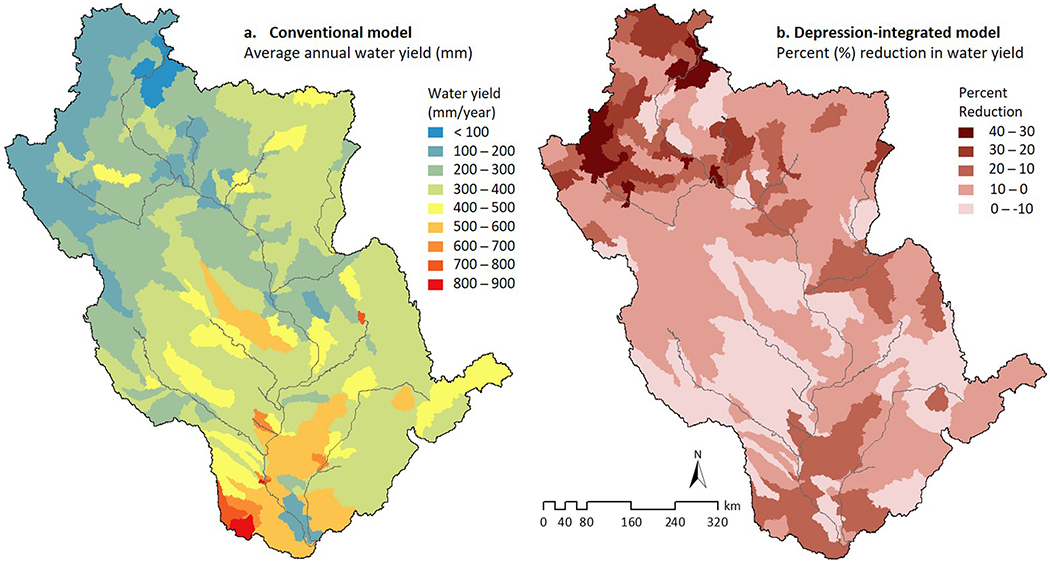
Effect of surface depressions on subbasin water yield: (a) 10-year average annual water yield (mm/year) simulated by the conventional “no-depression” model; (b) percent reduction in water yield due to the inclusion of surface depressions.

**Figure 4.**
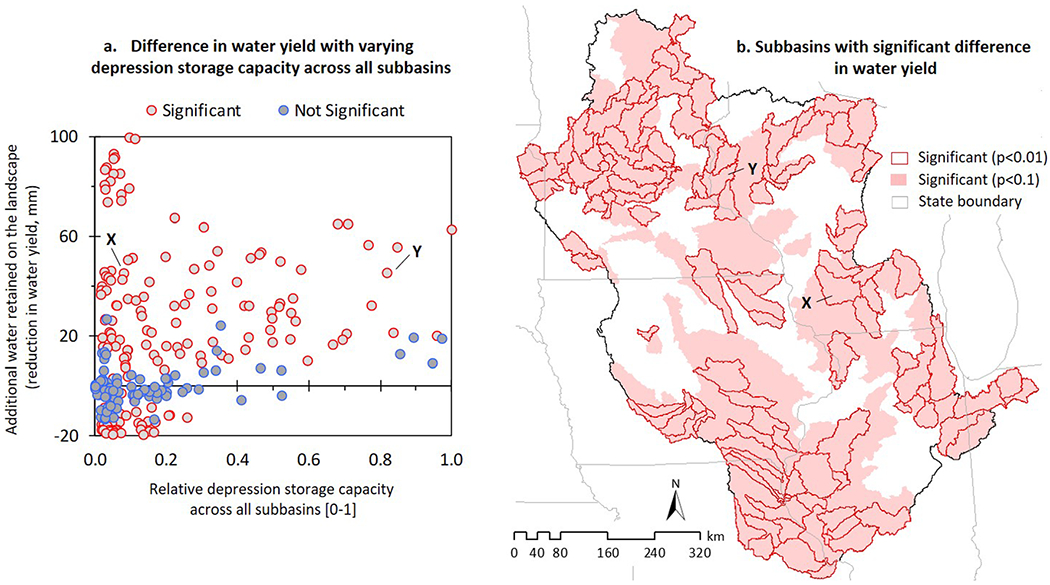
(a) Difference in water yields with varying surface depression water storage capacities across all subbasins. Each data point in this scatter-plot refers to a corresponding subbasin (*N* = 279); (b) subbasins with significant differences in water yield relative to the conventional model (significant at *p* < 0.1 level in 198 subbasins, out of which 111 were significant at *p* < 0.01). Surface depression storage capacity of a subbasin was estimated as the aggregated volume of all surface depressions existent therein. To express depression storage capacity using a uniform scale regardless of subbasin size, first the aggregated depression volume in each subbasin was converted into a volume per unit subbasin area (V/A) ratio; then this V/A ratio was normalized relative to the minimum and maximum V/A ratios across all subbasins. Subbasins X and Y are explicitly demarcated to show that substantially different depression storage capacities in two similarly sized subbasins (Figure S5) can have similar effects on respective water yield outputs.

**Figure 5.**
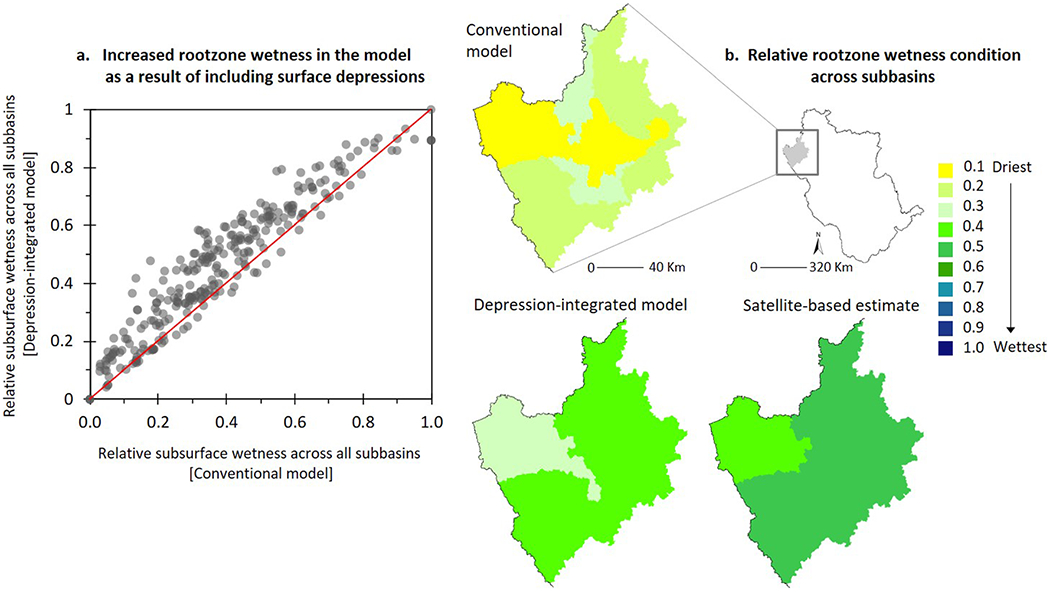
(a) Increased rootzone wetness in the depression-integrated model indicating a characteristic shift in water balance simulation compared to the conventional model. Each data point in this scatter-plot corresponds to a subbasin (*N* = 279). To address inconsistencies across subbasins in representing the rootzone and therefore allow a uniform comparison throughout the basin and potentially with satellite-based estimates, 10-year average annual simulated rootzone soil moisture content (mm) in each subbasin was normalized relative to the minimum and maximum soil moisture content across all subbasins; (b) spatial resemblance (bias) of subbasin rootzone wetness condition in the depression-integrated (conventional) model compared to the satellite-based estimates from Soil Moisture Active Passive (SMAP) mission. Values in the spatial maps are relative to the driest and wettest conditions throughout the UMRB.

In the subsequent sections, we therefore evaluate the additional differences in internal hydrologic dynamics of the two contrasting model configurations (conventional and depression integrated) focusing on landscape water yield (Figures 3 and 4) and rootzone wetness conditions (Figure 5).

### 3.2. Surface Depressions Alter Subbasin Water Yield Predictions

We found substantially different subbasin water yields between the conventional and depression-integrated configurations (Figure 3). Water yield, the amount of runoff generated from the landscape after accounting for all hydrologic losses (e.g., evapotranspiration and infiltration), is the most widely used index of water availability (e.g., Ellison et al., 2012; Fekete et al., 2002; Milly et al., 2005; Zhou et al., 2015). As previously noted, studies addressing water yield and availability often overlook the hydrologic effects of surface depressions (e.g., the conventional model configuration; Figure 3a). We therefore hypothesized that inclusion of surface depression storage would change water yield simulations both in their spatial patterns and subbasin-level volumes. Our depression-integrated UMRB model supported this hypothesis showing 10–40% reduction in subbasin (5% reduction in basin scale) annual water yields compared to the conventional model (Figure 3b). A visual analysis between Figures 1 and 3 indicates that the spatial density and associated storage volumes of surface depressions (Figure 1) correlate with the spatial variations in simulated water yields (Figure 3b). These results can also vary from year to year depending on the magnitude of precipitation (Figure S4).

Given our methodological approach, the variation in water yield between the two models stemmed solely from surface depressions (e.g., floodplain and non-floodplain wetlands, ponds, and other water storage systems). This is a new finding commonly overlooked in basin-scale/global water availability assessments (e.g., Liu et al., 2018; Veldkamp et al., 2017; Zhou et al., 2016). While Lehner et al. (2011) similarly suggested the considerable potential influence of small surface water storage systems on downstream waters, our study is, to our knowledge, the first to quantify this phenomenon in one of the world’s major river basins.

Simulated differences in water yield time-series between the two model configurations were statistically significant (*p* < 0.10) in ~70% of the basin area, which confirms the cumulative influence of surface depressions on basin-scale hydrologic dynamics (Figure 4a). However, additional evidence suggests that surface depression storage capacity (i.e., volume) alone does not fully explain their influence on downstream waters. Substantially different HEW volumes in two similarly sized subbasins can result in similar effects on the respective hydrologic responses at subbasin outlets. For example, in Figure 4a, two subbasins X and Y have similar drainage areas, yet the HEW volume is substantially larger in Y. The poorly drained soil and agricultural land use in subbasin Y suggests greater runoff potential compared to the well-drained soil and forested land use in subbasin X, but the smaller amount of precipitation in subbasin Y nullifies that potential. This, combined with the lower flow accumulation (milder slope) and less available non-depressional land (larger HEW volume), produces a runoff volume in subbasin Y that is too insufficient to fill the abundant depression storage therein (see, e.g., Nasab et al., 2017). As a result, even with different depression storage capacities, the model simulated reduction in water yields are similar for both subbasin X and Y because of the subbasins’ respective structural characteristics (see Figure S5).

To further demonstrate that factors in addition to storage volumes contribute to depressions’ effects on water yields across the UMRB, we mapped the subbasins where the depression-integrated model resulted in statistically significantly different water yield outputs compared to the conventional no-depression model (Figure 4b). Clearly, there are many subbasins where surface depressions do not have a significant effect on water yield despite their relatively substantive storage volumes compared to the rest of the basin (see, e.g., the abundance of depressions in west central UMRB; Figure 1) and vice versa (e.g., the relative dearth of depressions in south/southwest UMRB; Figure 1). Therefore, how significantly depression storage influences landscape water yield/availability depends on a combination of climatic and geophysical drivers (Thorslund et al., 2017).

### 3.3. Surface Depressions Improve Soil Moisture Accounting

The depression-integrated model generally retained more water on the landscape (Figure 4a). A simple justification for this response is that the runoff generated in the depression-integrated model had to fill an extra “water-storage bucket” (a HEW; on 2.3.3). This retention correspondingly led to increased soil moisture content in the rootzone (Figure 5a)—a fundamental process associated with the “sink-lag-source” functions of surface depressions (Lane et al., 2018; Rains et al., 2016; Rains, 2011). The correlation between surface depressions and subsurface wetness has also been recognized in recent studies (e.g., Tootchi et al., 2019). However, as we sought to calibrate the water balance by “curve-fitting” both model configurations with the same set of observed data, some subbasins had decreased water retention (hence, a decrease in rootzone moisture content) and more water runoff from the landscape despite the presence of depressions. We noted that this happened only in a few subbasins, particularly those with smaller HEW volumes (see, e.g., Figure 4a, lower left corner). Above a relative depression storage capacity of ~0.2, water is retained in the landscape. Consequently, retaining more water on the landscape and having a relatively wet rootzone was the characteristic, and anticipated, change in surface-subsurface hydrologic partitioning when depressions were included in the conventional model (Figure 5a). However, is this change one in the right direction, meaning does it improve the overall water balance in the model? We analyzed this issue using satellite-based SMAP soil moisture estimates (Figure 5b).

The spatial pattern of rootzone wetness condition derived from SMAP revealed a largely biased water balance in the conventional model (Figure 5b). Specifically, the conventional model simulated water balances such that subbasins in the northwest UMRB (as an example) were unrealistically “dry” (wetness index ~0.2) compared to the wettest (wetness index = 1) parts of the basin. This result occurred regardless of abundant depression storage in the basin. Those underrepresented dry subbasins in the conventional model appeared to be much wetter in the depression-integrated model and showed a strong spatial resemblance with SMAP estimates (wetness index ~0.4–0.5). These results confirm that including depression storage in conventional modeling practices can improve overall predictability of water balance and that these improvements happen for physically meaningful reasons.

## 4. Discussion

The inclusion of surface depressions in a properly parameterized hydrologic model will inevitably produce different—and as this study demonstrates, more accurate—predictions compared to the same model disregarding them. This should stimulate a reassessment of our conventional hydrologic modeling practices across large spatial domains. To gain further clarity on these issues, explore the implications of our findings, and therefore establish a meaningful rationale for depression-integrated hydrologic modeling, we address the following two questions:

1. What concepts are critical for interpreting the cumulative hydrologic effects of surface depressions in major river basins?
2. How would a depression-integrated model affect watershed management decisions?

### 4.1. What Concepts Are Critical for Interpreting the Cumulative Hydrologic Effects of Surface Depressions in Major River Basins?

To explain how surface depressions affect downstream waters, the degree of attenuation in high flow events has been the most widely acknowledged indicator (Blanchette et al., 2019; Huang et al., 2011; Shook & Pomeroy, 2011; Wilcox et al., 2011), although maintenance of baseflow is another important consideration (e.g., Evenson, Golden, et al., 2018). While peak flow attenuation helps prioritize land management alternatives via “what-if” scenarios focused on the abundance and spatial distribution of surface depressions (e.g., Ameli & Creed, 2019; Evenson, Golden, et al., 2018), we suggest that it cannot be a universal indicator for quantifying cumulative depression-effects. Our conjecture is based on the following “scale issues” (Blöschl & Sivapalan, 1995) not previously addressed in the literature.

**Figure 6.**
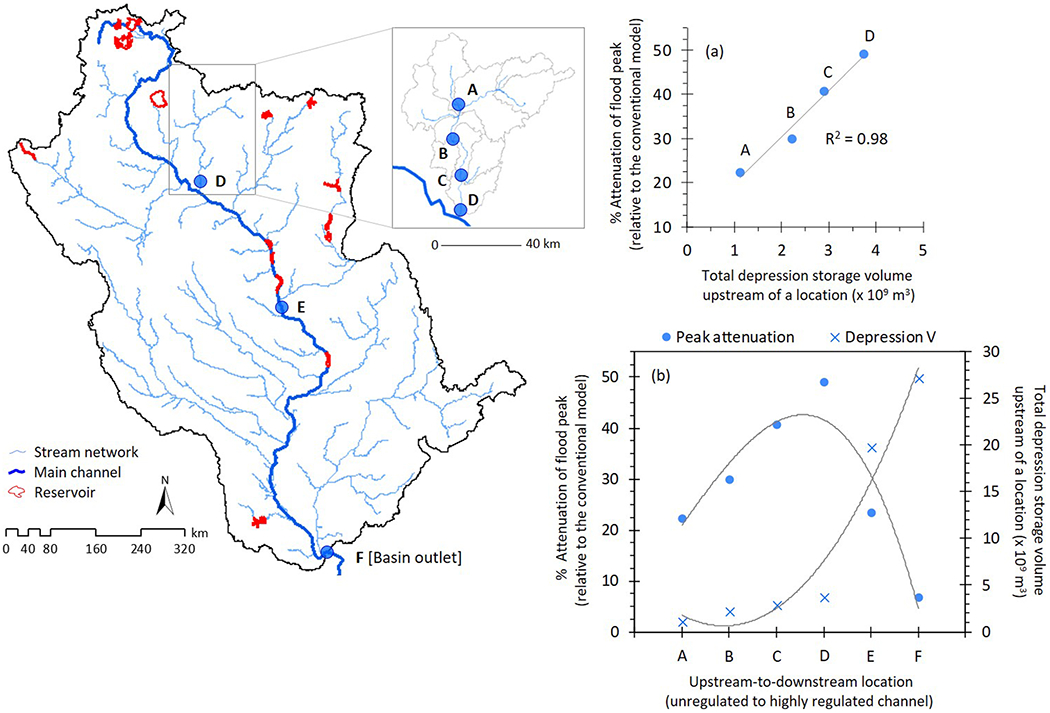
(a) Collinearity of peak flow attenuation with upstream abundance of depression storage volume in relatively unregulated headwater subbasins; (b) downstream dissipation of depressions’ peak flow attenuation capability due to flow regulations in a basin-scale stream network. Results are based on monthly average streamflow. To calculate % attenuation at every location (A-F), we considered their respective peak flows during 2008. We focused on 2008 because the largest historical flood across the entire length of our simulation occurred in this year (see Holmes et al., 2010).

First, the effect of surface depressions on peak flows is best understood in small- to meso-scale relatively unregulated watersheds (e.g., Blanchette et al., 2019; Nasab et al., 2017). In these systems, surface depressions typically demonstrate collinearity with peak flow conditions: As volumetric depression storage increases, peak flows exhibit greater attenuation (Figure 6a; also see Babbar-Sebens et al., 2013). It therefore may be expected that scaling these results further downstream and across much larger domains would result in peak flow attenuations that are proportional to the increased abundance of upstream surface depression storage. However, the converse may be true. The peak flow attenuation signal from surface depressions may dissipate when large gatekeeper lakes/reservoirs are present in the basin-scale river systems with regulated flow conditions (Figure 6b), along with numerous water withdrawal and point source discharge locations (Alexander et al., 2012; Graf, 2006; Zdankus et al., 2008). Our findings (Figure 6) confirm that spatial-scale effects on depressions’ peak flow attenuation capability is largely controlled by downstream flowpath characteristics (Quin et al., 2015), and as such, the local effects of an individual or a small group of surface depressions cannot be generalized over large spatial scales (Quin & Destouni, 2018; Thorslund et al., 2017).

Second, detecting peak flow attenuation through sub-daily or daily simulations almost invariably shows that the predominant downstream hydrologic effects come from large surface depression systems (e.g., Evenson, Golden, et al., 2018). Yet simulations across longer time scales (months, seasons, and years) may dampen the sub-daily and daily-simulated hydrologic signatures (Joo et al., 2018). As a result, the substantive effects from large depressions (e.g., areas with high HEW volumes in our UMRB model) represented by sub-daily or daily time scales may be insignificant across longer periods of assessment. For example, analyzing water yields using a 10-year average instead of sub-daily or daily time scale may be why some subbasins in the UMRB had relatively substantive depression storage yet a limited influence on water yield, and vice versa (Figure 4).

In this study, we spatially quantified the hydrologic changes between conventional and depression-integrated models at individual subbasin levels, instead of discrete locations across a basin-scale river network. Temporally, we used long-term average volume of water yield as the change indicator, instead of assessing peak flow attenuation in the rivers. This affords analyses of direct correlations between cause (depression storage volumes in subbasins) and effects (volumetric change in subbasin water yield), therefore explicitly informs where and how surface depressions alter hydrologic dynamics, and provides a basin-scale yet locally relevant interpretation.

### 4.2. How Would a Depression-Integrated Model Affect Watershed Management Decisions?

It is highly likely that studies disregarding surface depression storage in their models predict future floods to be more hazardous than they may be. Similarly, drought impacts on hydrologic processes in basins with surface depression storage will be over-predicted. We suggest this based on qualitatively comparing our results to some of the major modeling-based studies in the UMRB, the entire Mississippi Basin, and the continental United States in general. For example, multiple studies applying SWAT in the UMRB (Jha et al., 2004; Wu et al., 2012) have predicted a 50% increase in water yield for a 20% increase in the amount of precipitation, suggesting an amplification of future flood magnitudes. But the magnitude of variations in water yields between our two models indicates overpredicting flood hazards in models disregarding surface depressions (Figure 3). These discrepancies in model predictions may create imprecise and/or ineffective response strategies for flood and drought mitigation. Our findings suggest that model integration of surface depression would not only improve simulated accuracy in projected future flow regimes but provide a better informed approach for managing surface depressions as low-cost “nature-based solutions” (Bridgewater, 2018; Thorslund et al., 2017; Zalewski et al., 2018), in addition to the conventional structural measures (Kundzewicz et al., 2019; Tullos, 2018).

A key driver of regional water resources management from the overall water availability standpoint lies with our ability to delineate spatial locations of potential hydrologic changes. A multi-model comparison by Cai et al. (2014) acknowledged how some of the advanced atmospheric-land surface coupled models may misrepresent the spatial variability in hydrologic processes. Four basin/continental-scale models, including the Noah land surface model, Noah with multi-parameterization scheme (Noah-MP), Variable Infiltration Capacity (VIC), and Community Land Model-4 (CLM4)—models driving the widely used North American Land Data Assimilation System (NLDAS; NASA, 2019)—were configured by Cai et al. (2014) without surface depression storage. Results unanimously showed that water across the landscape was not in the right place. The authors demonstrated how simulated water balances, such as those in the depression-dominated northwest portion of UMRB, retained less water on the landscape compared to other parts of the basin. However, they also showed that remotely sensed terrestrial water storage anomalies (derived from Gravity Recovery and Climate Experiment, GRACE; Landerer & Swenson, 2012) revealed distinctively higher water storage capacity in the northwest UMRB compared to the rest of the basin—results that closely resemble the spatial abundance of depression storage we demonstrate here (Figure 1) and additional evidence supporting the higher rootzone wetness in our depression-integrated model (Figure 5, section 3.3).

It is therefore reasonable to argue that prior model-based studies designed to assist in spatially explicit watershed management may not be offering the best information to support sustainable decisions. Specifically, studies quantifying the relative contribution of upstream regions to downstream flow variability, separating out the role of climate and land use and attributing potential changes to local/regional driving factors (e.g., Du et al., 2018; Frans et al., 2013; Tao et al., 2014; Wise et al., 2017), may erroneously delineate targeted hydrologic management locations (e.g., Figure 4b) if surface depression storage is not considered in model simulations. This may lead to serious implications for socio-hydrologic modeling involving crop yield, irrigation requirements, and sustainable alternatives in agroecosystems to support human demand (Feng et al., 2018; Srinivasan et al., 2010; Yaeger et al., 2014).

In addition to the altered and improved hydrologic partitioning a depression-integrated model would provide (Figures 3 and 5), it would also activate and likely improve the modeled biogeochemical functions of surface depressions, such as their nutrient attenuation properties from settling, plant uptake, or losses to the atmosphere via denitrification. Golden et al. (2019) recently demonstrated this via a depression-integrated model, suggesting a 7% average decrease in annual nitrate yield from a ~17,000-km^2^ agricultural watershed compared to a conventional no-depression model. Using a depression-integrated modeling approach is particularly important for the UMRB because of its water quality problems resulting from intensive agricultural operations (Goolsby et al., 2000). Although hybrid models that integrate physical processes and data-driven statistical regression may simulate nutrient removal and decay in lakes and reservoirs (e.g., SPAtially Referenced Regression On Watershed (SPARROW) attributes model; Robertson & Saad, 2014), inclusion of millions of small depressional storage systems—even through a parsimonious approach (section 2.3.3)—would likely change existing water quality assessments. The overall changes in water quality projections from depression-integrated models would therefore likely impact environmental sustainability decisions (Fant et al., 2017; Li et al., 2016, 2017; Yen et al., 2016) including estimates of watershed-scale ecosystem services, applicable best management practices, and the economic consequences of both across the world’s major river basins (Golden et al., 2017).

## 5. Summary and Conclusions

We developed the first-ever major river basin-scale hydrologic model integrating surface depressions and focusing on the ~450,000-km^2^ Upper Mississippi River Basin (UMRB) in the United States. To do this, we first estimated the surface areas and storage capacities of ~455,000 surface depressions using a novel topography-based algorithm. We then integrated the aggregated depression areas and volumes at individual subbasins to most efficiently simulate depression hydrology across the UMRB. We conducted a 10-year simulation contrasting two model configurations (depression-integrated and conventional “no depression” models) and concluded the following:

1. Streamflow simulation accuracy increased in 54%, remained unchanged in 13%, and decreased in 33% of the target locations as a result of including surface depression storage. The relative improvement of streamflow simulation accuracy at target stream locations correlated with the upstream abundance of depression storage, indicating enhanced process representation in the depression-integrated model.
2. Subbasin water yields from the depression-integrated model significantly altered UMRB’s hydrologic response compared to the conventional model across 70% of the basin area (~315,000 km^2^). This significant variation was additive after accounting for major lakes and reservoirs typically considered in conventional modeling practices.
3. A characteristic shift in the partitioning of hydrologic fluxes due to the inclusion of surface depressions produced a physically realistic water balance. Specifically, the depression-integrated model generally increased the overall wetness of the rootzone, showing strong spatial resemblance with satellite-based estimates.

While these results suggested improved hydrologic prediction by a depression-integrated model, we performed additional analyses to demonstrate that substantially different depression storage capacities (i.e., volumes) in similarly sized subbasins can impose similar effects on the respective hydrologic responses at subbasin outlets. To fully understand where and how surface depressions may be influential, it is therefore necessary to consider how climatic and geophysical drivers interact with the presence of surface depressions on the landscape.

We also highlighted conceptual issues, specifically how the spatial and temporal scale of analyses affect—and may impede—interpretations of depressions’ cumulative hydrologic effects on downstream waters. For example, we analyzed how surface depression storage attenuated peak flows in the UMRB, and we concluded that unlike small watersheds, flowpath regulation in large river basins (i.e., gatekeeper reservoirs and dams) is a major factor affecting the quantification of cumulative depression-effects. Therefore, our results suggest that the localized hydrologic effects of an individual depression or small group of depressions cannot be generalized across basin scales.

The findings presented in this study stimulate the need for re-envisioning our conventional hydrologic modeling practices. Yet such re-envisioning would require addressing the big data challenges associated with depression-integrated modeling. Recent developments of globally accessible, sufficiently high-resolution waterbody maps, efficient algorithms to retrieve depression area and volume from hyper-resolution topographic data, and tools for seamless data-model integration across any spatial scale of interest will advance depression-integrated modeling by improving the physical representation of the landscape and quantification of surface depression storage. The next challenge is to use these data in the most computationally efficient manner to propel the hydro-geoscience community closer to depression-integrated hydrologic modeling as the “new normal.”

## Supplementary Material

Supplement1

## Figures and Tables

**Table 1:** Input Data Sets for the Upper Mississippi River Basin SWAT Model

Data type	Description	Reference(s)
Topography	90-m NED (resampled from 30 m)	USGS (2018a)
Land use	30-m 2011 CDL	NASS (2018)
Soil texture	1:250,000 STATSGO built-in with SWAT database	NRCS (2018)
Weather forcing	Total daily precipitation, minimum, and maximum daily temperature from 1-km gridded Daymet product	Thornton et al. (2018)
	Solar radiation, wind speed, and relative humidity from SWAT’s built-in historical database (weather generator)	Neitsch et al. (2011)
Subsurface drainage for poorly drained soils	30-m tile drain extent data for the Midwestern United States, derived using 2011 NLCD land cover and STATSGO soil database (Figure S1)	Nakagaki and Wieczorek (2016)
Reservoir storage-discharge	15 major reservoirs; identified as Level-1 waterbodies in the Global Lakes and Wetlands database (Figure 2a)	Lehner and Döll (2004)
	Storage-discharge parameter values for individual reservoirs; daily dam outflow data at five locations (Figure S2)	USACE (2018)
Surface depressions^a^	Surface depressions identified from the 30-m NED; depression area and volume were estimated using a topography-based algorithm	Wu et al. (2019) and Wu and Lane (2016)
Gage station streamflow	30 USGS gage stations for model calibration and verification (Figures 2a and S3)	USGS (2018b)
Satellite-based soil moisture^b^	SMAP mission L4 global 9-km rootzone soil moisture; 3-hourly estimates averaged into monthly time scale and aggregated at subbasin level; used for model cross-verification	Reichle et al. (2018)

Abbreviations: NED = National Elevation Data Set; USGS = U.S. Geological Survey; CDL = cropland data layer; NASS = National Agricultural Statistics Service; STATSGO = State Soil Geographic Database; NLCD = National Land Cover Database; USACE = U.S. Army Corps of Engineers; SMAP = Soil Moisture Active Passive.

a Required only in the *depression-integrated* model configuration (see details in sections 2.3.2 and 2.3.3).

b Using SMAP data for the entire simulation period (2008–2017) was not feasible because of data availability since 31 March 2015. Specifically, we used SMAP data for one crop growing season (1 June–31 August 2016) because this is the most “active” period in terms of vegetation growth and subsequent soil moisture dynamics. Further, data obtained for this period are not affected by snow cover and frozen soil (Reichle et al., 2017).

## Key Points:

- We present the first hydrologic model incorporating surface depression and wetland water storage in one of the world’s major river basins
- Surface depressions, often overlooked in conventional large-scale hydrologic modeling, significantly alter landscape water yields
- Cumulative hydrologic effects of surface depression storage on downstream waters depend on a combination of local drivers

## References

[R1] Abbaspour KC, Rouholahnejad E, Vaghefi S, Srinivasan R, Yang H, & Kløve B (2015). A continental-scale hydrology and water quality model for Europe: Calibration and uncertainty of a high-resolution large-scale SWAT model. Journal of Hydrology, 524,733–752. 10.1016/j.jhydrol.2015.03.027

[R2] Abbaspour KC (2015). User manual for SWAT-CUP, SWAT calibration, and uncertainty analysis programs. https://swat.tamu.edu/media/114860/usermanual_swatcup.pdf, Accessed: August 28, 2019.

[R3] Aires F, Miolane L, Prigent C, Pham B, Fluet-Chouinard E, Lehner B, & Papa F (2017). A global dynamic long-term inundation extent dataset at high spatial resolution derived through downscaling of satellite observations. Journal of Hydrometeorology, 18(5), 1305–1325. 10.1175/JHM-D-16-0155.1

[R4] Alexander JS, Wilson RC, & Green WR (2012). A brief history and summary of the effects of river engineering and dams on the Mississippi River system and delta. U.S. Geological Survey Circular 1375 https://pubs.usgs.gov/circ/1375/C1375.pdf

[R5] Alexander LC, Fritz KM, Schofield KA, Autrey BC, DeMeester JE, Golden HE, (2018). Featured collection introduction: Connectivity of streams and wetlands to downstream waters. JAWRA Journal of the American Water Resources Association, 54(2), 287–297. 10.1111/1752-1688.12630

[R6] Almendinger JE, Murphy MS, & Ulrich JS (2014). Use of the soil and water assessment tool to scale sediment delivery from field to watershed in an agricultural landscape with topographic depressions. Journal of Environmental Quality, 43(1), 9–17. 10.2134/jeq2011.0340 25602535

[R7] Ameli AA, & Creed IF (2017). Quantifying hydrologic connectivity of wetlands to surface water systems. Hydrology and Earth System Sciences, 21(3), 1791–1808. 10.5194/hess-21-1791-2017

[R8] Ameli AA, & Creed IF (2019). Does wetland location matter when managing wetlands for watershed-scale flood and drought resilience? JAWRA Journal of the American Water Resources Association, 55(3), 529–542. 10.1111/1752-1688.12737

[R9] Arnold JG, Moriasi DN, Gassman PW, Abbaspour KC, White M, Srinivasan R, (2012). SWAT: Model use, calibration,and validation. Transactions of the ASABE, 55(4), 1491–1508. 10.13031/2013.42256

[R10] Babbar-Sebens M, Barr RC, Tedesco LP, & Anderson M (2013). Spatial identification and optimization of upland wetlands in agricultural watersheds. Ecological Engineering, 52, 130–142. 10.1016/j.ecoleng.2012.12.085

[R11] Bauer P, Thorpe A, & Brunet G (2015). The quiet revolution of numerical weather prediction. Nature, 525(7567), 47–55. 10.1038/nature14956 26333465

[R12] Beven KJ, & Cloke HL (2012). Comment on “hyperresolution global land surface modeling: Meeting a grand challenge for monitoring Earth’s terrestrial water” by Eric F. Wood et al. Water Resources Research, 48(1). 10.1029/2011WR010982

[R13] Bierkens MFP, Bell VA, Burek P, Chaney N, Condon LE, David CH, (2015). Hyper-resolution global hydrological modelling: What is next? Hydrological Processes, 29(2), 310–320. 10.1002/hyp.10391

[R14] Biggs J, von Fumetti S, & Kelly-Quinn M (2017). The importance of small waterbodies for biodiversity and ecosystem services: Implications for policy makers. Hydrobiologia, 793(1), 3–39. 10.1007/s10750-016-3007-0

[R15] Blanchette M, Rousseau AN, Foulon E, Savary S, & Poulin M (2019). What would have been the impacts of wetlands on low flow support and high flow attenuation under steady state land cover conditions? Journal of Environmental Management, 234, 448–457. 10.1016/j.jenvman.2018.12.095 30640170

[R16] Blöschl G, & Sivapalan M (1995). Scale issues in hydrological modelling: A review. Hydrological Processes, 9(3–4), 251–290. 10.1002/hyp.3360090305

[R17] Bridgewater P (2018). Whose nature? What solutions? Linking ecohydrology to nature-based solutions. Ecohydrology & Hydrobiology, 18(4), 311–316. 10.1016/j.ecohyd.2018.11.006

[R18] Brooks JR, Mushet DM, Vanderhoof MK, Leibowitz SG, Christensen JR, Neff BP, (2018). Estimating wetland connectivity to streams in the prairie pothole region: An isotopic and remote sensing approach. Water Resources Research, 54(2), 995–977. 10.1002/2017WR021016 29681665 PMC5903587

[R19] Cai X, Yang ZL, Xia Y, Huang M, Wei H, Leung LR, & Ek MB (2014). Assessment of simulated water balance from Noah, Noah-MP, CLM, and VIC over conus using the NLDAS test bed. Journal of Geophysical Research: Atmospheres, 119, 13,751–13,770. 10.1002/2014JD022113

[R20] Camporese M, Daly E, & Paniconi C (2015). Catchment-scale Richards equation-based modeling of evapotranspiration via boundary condition switching and root water uptake schemes. Water Resources Research, 51, 5756–5771. 10.1002/2015WR017139

[R21] Chen F, Crow WT, Cosh MH, Colliander A, Asanuma J, Berg A, (2019). Uncertainty of reference pixel soil moisture averages sampled at SMAP core validation sites. Journal of Hydrometeorology, 20(8), 1553–1569. 10.1175/JHM-D-19-0049.1

[R22] Chu X (2017). Delineation of pothole-dominated wetlands and modeling of their threshold behaviors. Journal of Hydrologic Engineering, 22(1), D5015003 10.1061/(ASCE)HE.1943-5584.0001224

[R23] Clark MP, Bierkens MFP, Samaniego L, Woods RA, Uijlenhoet R, Bennett KE, (2017). The evolution of process-based hydrologic models: Historical challenges and the collective quest for physical realism. Hydrology and Earth System Sciences, 21(7), 3427–3440. 10.5194/hess-21-3427-2017 32747855 PMC7398150

[R24] Clark MP, Fan Y, Lawrence DM, Adam JC, Bolster D, Gochis DJ, (2015). Improving the representation of hydrologic processes in Earth System models. Water Resources Research, 51(8), 5929–5956. 10.1002/2015WR017096

[R25] Cohen MJ, Creed IF, Alexander L, Basu NB, Calhoun AJK, Craft C, (2016). Do geographically isolated wetlands influence landscape functions? Proceedings of the National Academy of Sciences, 113(8), 1978–1986. 10.1073/pnas.1512650113 PMC4776504 26858425

[R26] Creed IF, Lane CR, Serran JN, Alexander LC, Basu NB, Calhoun AJK, (2017). Enhancing protection for vulnerable waters. Nature Geoscience, 10(11), 809–815. 10.1038/ngeo3041 PMC6071434 30079098

[R27] Dang TD, Chowdhury AFMK, & Galelli S (2020). On the representation of water reservoir storage and operations in large-scale hydrological models: Implications on model parameterization and climate change impact assessments. Hydrology and Earth System Sciences, 24(1), 397–416. 10.5194/hess-24-397-2020

[R28] Downing JA (2010). Emerging global role of small lakes and ponds: Little things mean a lot. Limnetica, 29(1), 9–24.

[R29] Du L, Rajib A, & Merwade V (2018). Large scale spatially explicit modeling of blue and green water dynamics in a temperate mid-latitude basin. Journal of Hydrology, 562, 84–102. 10.1016/j.jhydrol.2018.02.071

[R30] Ellison D, Futter MN, & Bishop K (2012). On the forest cover-water yield debate: From demand- to supply-side thinking. Global Change Biology, 18(3), 806–820. 10.1111/j.1365-2486.2011.02589.x

[R31] Endrizzi S, Gruber S, Dall’Amico M, & Rigon R (2014). GEOtop 2.0: Simulating the combined energy and water balance at and below the land surface accounting for soil freezing, snow cover and terrain effects. Geoscientific Model Development, 7(6), 2831–2857. 10.5194/gmd-7-2831-2014

[R32] Evenson GR, Golden HE, Lane CR, & D’Amico E (2016). An improved representation of geographically isolated wetlands in a watershed-scale hydrologic model. Hydrological Processes, 30(22), 4168–4184. 10.1002/hyp.10930

[R33] Evenson GR, Golden HE, Lane CR, McLaughlin DL, & D’Amico E (2018). Depressional wetlands affect watershed hydrological, biogeochemical, and ecological functions. Ecological Applications, 28(4), 953–966. 10.1002/eap.1701 29437239 PMC7724629

[R34] Evenson GR, Jones CN, McLaughlin DL, Golden HE, Lane CR, DeVries B, (2018). A watershed-scale model for depressional wetland-rich landscapes. Journal of Hydrology X, 1, 100002 10.1016/j.hydroa.2018.10.002 PMC6707518 31448367

[R35] Fant C, Srinivasan R, Boehlert B, Rennels L, Chapra SC, Strzepek KM, (2017). Climate change impacts on US water quality using two models: HAWQS and US basins. Watermark, 9(2), 118 10.3390/w9020118

[R36] Faramarzi M, Abbaspour KC, Adamowicz WLV, Lu W, Fennell J, Zehnder AJB, & Goss GG (2017). Uncertainty based assessment of dynamic freshwater scarcity in semi-arid watersheds of Alberta, Canada. Journal of Hydrology: Regional Studies, 9,48–68. 10.1016/j.ejrh.2016.11.003

[R37] Fatichi S, Vivoni ER, Ogden FL, Ivanov VY, Mirus B, Gochis D, (2016). An overview of current applications, challenges, and future trends in distributed process-based models in hydrology. Journal of Hydrology, 537, 45–60. 10.1016/j.jhydrol.2016.03.026

[R38] Fekete BM, Vörösmarty CJ, & Grabs W (2002). High-resolution fields of global runoff combining observed river discharge and simulated water balances. Global Biogeochemical Cycles, 16(3), 15-1–15-10. 10.1029/1999GB001254

[R39] Feng Q, Chaubey I, Cibin R, Engel B, Sudheer KP, Volenec J, & Omani N (2018). Perennial biomass production from marginal land in the Upper Mississippi River Basin. Land Degradation and Development, 29(6), 1748–1755. 10.1002/ldr.2971

[R40] Feng XQ, Zhang GX, & Jun Xu Y (2013). Simulation of hydrological processes in the Zhalong wetland within a river basin, Northeast China. Hydrology and Earth System Sciences, 17(7), 2797–2807. 10.5194/hess-17-2797-2013

[R41] Fossey M, & Rousseau AN (2016). Can isolated and riparian wetlands mitigate the impact of climate change on watershed hydrology? A case study approach. Journal of EnvironmentalManagement, 184(Pt 2), 327–339. 10.1016/j.jenvman.2016.09.043 27745769

[R42] Fossey M, Rousseau AN, Bensalma F, Savary S, & Royer A (2015). Integrating isolated and riparian wetland modules in the PHYSITEL/HYDROTEL modelling platform: Model performance and diagnosis. Hydrological Processes, 29(22), 4683–4702. 10.1002/hyp.10534

[R43] Frans C, Istanbulluoglu E, Mishra V, Munoz-Arriola F, & Lettenmaier DP (2013). Are climatic or land cover changes the dominant cause of runoff trends in the Upper Mississippi River Basin? Geophysical Research Letters, 40(6), 1104–1110. 10.1002/grl.50262

[R44] Gao H (2015). Satellite remote sensing of large lakes and reservoirs: From elevation and area to storage. Wiley Interdisciplinary Reviews Water, 2(2), 147–157. 10.1002/wat2.1065

[R45] Gao H, Birkett C, & Lettenmaier DP (2012). Global monitoring of large reservoir storage from satellite remote sensing. Water Resources Research, 48(9). 10.1029/2012WR012063

[R46] Gasper F, Goergen K, Shrestha P, Sulis M, Rihani J, Geimer M, & Kollet S (2014). Implementation and scaling of the fully coupled terrestrial systems modeling platform (TerrSysMP v1.0) in a massively parallel supercomputing environment—A case study on JUQUEEN (IBM Blue Gene/Q). Geoscientific Model Development, 7(5), 2531–2543. 10.5194/gmd-7-2531-2014

[R47] Gassman PW, Reyes MR, Green CH, & Arnold JG (2007). SWAT: Hystorical development, applications, and future research directions. Transactions of the ASABE, 50(4), 1211–1250. 10.13031/2013.23637

[R48] Gleason RA, Tangen BA, Laubhan MK, Kermes KE, & Euliss NH Jr (2007). Estimating water storage capacity of existing and potentially restorable wetland depressions in a subbasin of the Red River of the North. USGS report 2007-1159. 10.3133/ofr20071159

[R49] Golden HE, Creed IF, Ali G, Basu NB, Neff BP, Rains MC, (2017). Integrating geographically isolated wetlands into land management decisions. Frontiers in Ecology and the Environment, 15(6), 319–327. 10.1002/fee.1504 30505246 PMC6261316

[R50] Golden HE, Lane CR, Amatya DM, Bandilla KW, Raanan Kiperwas H, Knightes CD, & Ssegane H (2014). Hydrologic connectivity between geographically isolated wetlands and surface water systems: A review of select modeling methods. Environmental Modelling & Software, 53, 190–206. 10.1016/j.envsoft.2013.12.004

[R51] Golden HE, Rajib A, Lane CR, Christensen JR, Wu Q, & Mengistu S (2019). Non-floodplain wetlands affect watershed nutrient dynamics: A critical review. Environmental Science & Technology, 53(13), 7203–7214. 10.1021/acs.est.8b07270 31244063 PMC9096804

[R52] Golden HE, Sander HA, Lane CR, Zhao C, Price K, D’Amico E, & Christensen JR (2016). Relative effects of geographically isolated wetlands on streamflow: A watershed-scale analysis. Ecohydrology, 9(1), 21–38. 10.1002/eco.1608

[R53] Goolsby DA, Battaglin WA, Aulenbach BT, & Hooper RP (2000). Nitrogen flux and sources in the Mississippi River Basin. Science of the Total Environment, 248(2–3), 75–86. 10.1016/S0048-9697(99)00532-X 10805229

[R54] Graf WL (2006). Downstream hydrologic and geomorphic effects of large dams on American rivers. Geomorphology, 79(3–4), 336–360. 10.1016/j.geomorph.2006.06.022

[R55] Gupta HV, Kling H, Yilmaz KK, & Martinez GF (2009). Decomposition of the mean squared error and NSE performance criteria: Implications for improving hydrological modelling. Journal of Hydrology, 377, 80–91. 10.1016/j.jhydrol.2009.08.003

[R56] Holmes RR Jr., Koenig TA, & Karstensen KA (2010). Flooding in the United States Midwest, 2008. U.S. Geological Survey Professional Paper 1775 https://pubs.usgs.gov/pp/1775/pdf/pp1775.pdf

[R57] Huang S, Young C, Feng M, Heidemann K, Cushing M, Mushet DM, & Liu S (2011). Demonstration of a conceptual model for using LiDAR to improve the estimation of floodwater mitigation potential of prairie pothole region wetlands. Journal of Hydrology, 405(3–4), 417–426. 10.1016/j.jhydrol.2011.05.040

[R58] Hutchinson KJ, & Christiansen DE (2013). Use of the soil and water assessment tool (SWAT) for simulating hydrology and water quality in the Cedar River Basin, Iowa, 2000–10. U.S. Geological Survey Scientific Investigations Report 2013–5002 https://pubs.usgs.gov/sir/2013/5002/

[R59] Jan A, Coon ET, Graham JD, & Painter SL (2018). A subgrid approach for modeling microtopography effects on overland flow. Water Resources Research, 54(9), 6153–6167. 10.1029/2017WR021898

[R60] Jha M, Pan Z, Takle E, & Roy G (2004). Impacts of climate change on streamflow in the Upper Mississippi River Basin: A regional climate model perspective. Journal of Geophysical Research, 109, D09105 10.1029/2003JD003686

[R61] Jones CN, Ameli A, Neff BP, Evenson GR, McLaughlin DL, Golden HE, & Lane CR (2019). Modeling connectivity of non-floodplain wetlands: Insights, approaches, and recommendations. JAWRA Journal of the American Water Resources Association, 55(3), 559–577. 10.1111/1752-1688.12735 PMC8312621 34316250

[R62] Jones CN, Evenson GR, McLaughlin DL, Vanderhoof MK, Lang MW, McCarty GW, (2017). Estimating restorable wetland water storage at landscape scales. Hydrological Processes, 32(2), 305–313. 10.1002/hyp.11405 PMC5907502 29681686

[R63] Joo J, Tian Y, Zheng C, Zheng Y, Sun Z, Zhang A, & Chang H (2018). An integrated modeling approach to study the surface water-groundwater interactions and influence of temporal damping effects on the hydrological cycle in the Miho catchment in South Korea. Watermark, 10(11), 1529 10.3390/w10111529

[R64] Kauffeldt A, Halldin S, Rodhe A, Xu C-Y, & Westerberg IK (2013). Disinformative data in large-scale hydrological modelling. Hydrology and Earth System Sciences, 17(7), 2845–2857. 10.5194/hess-17-2845-2013

[R65] Kirchner JW (2006). Getting the right answers for the right reasons: Linking measurements, analyses, and models to advance the science of hydrology. Water Resources Research, 42(3). 10.1029/2005WR004362

[R66] Knoben WJM, Freer J, & Woods RA (2019). Technical note: Inherent benchmark or not? Comparing Nash–Sutcliffe and Kling–Gupta efficiency scores. Hydrology and Earth System Sciences, 23(10), 4323–4331. 10.5194/hess-23-4323-2019

[R67] Kollet SJ, & Maxwell RM (2008). Capturing the influence of groundwater dynamics on land surface processes using an integrated, distributed watershed model. Water Resources Research, 44(2). 10.1029/2007WR006004

[R68] Koster RD, Liu Q, Mahanama SPP, & Reichle RH (2018). Improved hydrological simulation using SMAP data: Relative impacts of model calibration and data assimilation. Journal of Hydrometeorology, 19(4), 727–741. 10.1175/JHM-D-17-0228.1 29983646 PMC6031932

[R69] KSU Libraries (2017). Paired samples t test. Kent State University Available online at: http://libguides.library.kent.edu/SPSS/PairedSamplestTest Accessed: April 28, 2019

[R70] Kundzewicz ZW, Hegger DLT, Matczak P, & Driessen PPJ (2019). Opinion: Flood-risk reduction: Structural measures and diverse strategies. Proceedings of the National Academy of Sciences, 115(49), 12,321–12,325. 10.1073/pnas.1818227115 PMC6298124 30514751

[R71] Landerer FW, & Swenson SC (2012). Accuracy of scaled GRACE terrestrial water storage estimates. Water Resources Research, 48(4). 10.1029/2011WR011453

[R72] Lane CR, Leibowitz SG, Autrey BC, LeDuc SD, & Alexander LC (2018). Hydrological, physical, and chemical functions and connectivity of non-floodplain wetlands to downstream waters: A review. JAWRA Journal of the American Water Resources Association, 54(2), 346–371. 10.1111/1752-1688.12633 PMC8654163 34887654

[R73] Lee S, Yeo I-Y, Lang MW, Sadeghi AM, McCarty GW, Moglen GE, & Evenson GR (2018). Assessing the cumulative impacts of geographically isolated wetlands on watershed hydrology using the SWAT model coupled with improved wetland modules. Journal of Environmental Management, 223, 37–48. 10.1016/j.jenvman.2018.06.006 29886149

[R74] Lehner B, & Doll P (2004). Development and validation of a global database of lakes, reservoirs and wetlands. Journal of Hydrology, 296(1–4), 1–22. 10.1016/johydrol.2004.03.028

[R75] Lehner B, Liermann CR, Revenga C, Vörömsmarty C, Fekete B, Crouzet P, (2011). High-resolution mapping of the world’s reservoirs and dams for sustainable river-flow management. Frontiers in Ecology and the Environment, 9(9), 494–502. 10.1890/100125

[R76] Leibowitz SG, Wigington PJ, Rains MC, & Downing DM (2008). Non-navigable streams and adjacent wetlands: Addressing science needs following the Supreme Court’s Rapanos decision. Frontiers in Ecology and the Environment, 6(7), 364–371. 10.1890/070068

[R77] Li P, Chaubey I, Muenich RL, & Wei X (2016). Evaluation of fresh water provisioning for different ecosystem services in the Upper Mississippi River Basin: Current status and drivers. Watermark, 8(7). 10.3390/W8070288

[R78] Li P, Omani N, Chaubey I, & Wei X (2017). Evaluation of drought implications on ecosystem services: Freshwater provisioning and food provisioning in the Upper Mississippi River Basin. International Journal of Environmental Research and Public Health, 14(5). 10.3390/ijerph14050496 PMC5451947 28481311

[R79] Liu L, Parkinson S, Gidden M, Byers E, Satoh Y, Riahi K, & Forman B (2018). Quantifying the potential for reservoirs to secure future surface water yields in the world’s largest river basins. Environmental Research Letters, 13(4), 044026 10.1088/1748-9326/aab2b5

[R80] Liu Y, Yang W, & Wang X (2008). Development of a SWAT extension module to simulate riparian wetland hydrologic processes at a watershed scale. Hydrological Processes, 22(16), 2901–2915. 10.1002/hyp.6874

[R81] Maxwell RM, Condon LE, & Kollet SJ (2015). A high-resolution simulation of groundwater and surface water over most of the continental US with the integrated hydrologic model ParFlow v3. Geoscientific Model Development, 8(3), 923–937. 10.5194/gmd-8-923-2015

[R82] McLaughlin DL, Kaplan DA, & Cohen MJ (2014). A significant nexus: Geographically isolated wetlands influence landscape hydrology. Water Resources Research, 50, 7153–7166. 10.1002/2013WR015002

[R83] Milly PCD, Dunne KA, & Vecchia AV (2005). Global pattern of trends in streamflow and water availability in a changing climate. Nature, 438(7066), 347–350. 10.1038/nature04312 16292308

[R84] Moriasi DN, Rossi CG, Arnold JG, & Tomer MD (2012). Evaluating hydrology of the soil and water assessment tool (SWAT) with new tile drain equations. Journal of Soil and Water Conservation, 67(6), 513–524. 10.2489/jswc.67.6.513

[R85] Nakagaki N, & Wieczorek ME (2016). Estimates of subsurface tile drainage extent for 12 Midwest states, 2012. U.S. Geological Survey data release. 10.5066/F7W37TDP

[R86] NASA (2019). README document for North American Land Data Assimilation System Phase 2 (NLDAS-2) Products. Goddard Earth Sciences Data and Information Services Center. National Aeronautics and Space Administration https://hydro1.gesdisc.eosdis.nasa.gov/data/NLDAS/README.NLDAS2.pdf Accessed: June 28, 2019

[R87] Nasab MT, & Chu X (2020). Macro-HyProS: A new macro-scale hydrologic processes simulator for depression-dominated cold climate regions. Journal of Hydrology, 580, 124366 10.1016/j.jhydrol.2019.124366

[R88] Nasab MT, Singh V, & Chu X (2017). SWAT modeling for depression-dominated areas: How do depressions manipulate hydrologic modeling? Watermark, 9(1), 58 10.3390/w9010058

[R89] NASS (2018). Cropland data layer. National Agricultural Statistics Service. U.S. Department of Agriculture https://nassgeodata.gmu.edu/CropScape/ Accessed: April 3, 2019

[R90] Neitsch SL, Arnold JG, Kiniry JR, & Williams JR (2011). Soil & water assessment tool theoretical documentation version 2009. Texas Water Resources Institute Technical Report No. 406 https://swat.tamu.edu/media/99192/swat2009-theory.pdf

[R91] NRCS (2018). Web Soil Survey Natural resources conservation service. U.S. Department of Agriculture https://websoilsurvey.nrcs.usda.gov/

[R92] Pekel J-F, Cottam A, Gorelick N, & Belward AS (2016). High-resolution mapping of global surface water and its long-term changes. Nature, 540(7633), 418–422. 10.1038/nature20584 27926733

[R93] Pokhrel Y, Shin S, Lin Z, Yamazaki D,& Qi J (2018). Potential disruption of flood dynamics in the lower Mekong River basin due to upstream flow regulation. Scientific Reports, 8(1), 17767 10.1038/s41598-018-35823-4 30532063 PMC6288158

[R94] Prudhomme C, Giuntoli I, Robinson EL, Clark DB, Arnell NW, Dankers R, (2014). Hydrological droughts in the 21st century, hotspots and uncertainties from a global multimodel ensemble experiment. Proceedings of the National Academy of Sciences, 111(9), 3262–3267. 10.1073/pnas.1222473110 PMC3948235 24344266

[R95] Quin A, & Destouni G (2018). Large-scale comparison of flow-variability dampening by lakes and wetlands in the landscape. Land Degradation and Development, 29(10), 3617–3627. 10.1002/ldr.3101

[R96] Quin A, Jaramillo F, & Destouni G (2015). Dissecting the ecosystem service oflarge-scale pollutant retention: The role ofwetlands and other landscape features. Ambio, 44(1), 127–137. 10.1007/s13280-014-0594-8 PMC4288994 25576287

[R97] Quinn N, Bates PD, Neal J, Smith A, Wing O, Sampson C, (2019). The spatial dependence of flood Hazard and risk in the United States. Water Resources Research, 55(3), 1890–1911. 10.1029/2018WR024205

[R98] Rahman MM, Thompson JR, & Flower RJ (2016). An enhanced SWAT wetland module to quantify hydraulic interactions between riparian depressional wetlands, rivers and aquifers. Environmental Modelling and Software, 84, 263–289. 10.1016/j.envsoft.2016.07.003

[R99] Rains M, Leibowitz S, Cohen M, Creed I, Golden H, Jawitz J, (2016). Geographically isolated wetlands are part of the hydrological landscape. Hydrological Processes, 30(1), 153–160. 10.1002/hyp.10635

[R100] Rains MC (2011). Water sources and hydrodynamics of closed-basin depressions, Cook Inlet Region, Alaska. Wetlands, 31(2), 377–387. 10.1007/s13157-011-0147-x

[R101] Rajib A, Evenson GR, Golden HE, & Lane CR (2018). Hydrologic model predictability improves with spatially explicit calibration using remotely sensed evapotranspiration and biophysical parameters. Journal of Hydrology, 567, 668–683. 10.1016/j.jhydrol.2018.10.024 31395990 PMC6687302

[R102] Rajib A, Liu Z, Merwade V, Tavakoly AA, & Follum ML (2020). Towards a large-scale locally relevant flood inundation modeling framework using SWAT and LISFLOOD-FP. Journal of Hydrology, 581,124406 10.1016/j.jhydrol.2019.124406

[R103] Rajib A, & Merwade V (2017). Hydrologic response to future land use change in the Upper Mississippi River Basin by the end of 21st century. Hydrological Processes, 31(21), 3645–3661. 10.1002/hyp.11282

[R104] Rajib A, Merwade V, Kim IL, Zhao L, Song C, & Zhe S (2016). SWATShare—A web platform for collaborative research and education through online sharing, simulation and visualization of SWAT models. Environmental Modelling & Software, 75,498–512. 10.1016/j.envsoft.2015.10.032

[R105] Rajib A, Merwade V, & Yu Z (2018). Rationale and efficacy of assimilating remotely sensed potential evapotranspiration for reduced uncertainty of hydrologic models. Water Resources Research, 54(7), 4615–4637. 10.1029/2017WR021147

[R106] Reichle R, De Lannoy G, Koster RD, Crow W, Kimball J, & Liu Q (2018). SMAP L4 Global 3-hourly 9 km EASE-grid surface and root zone soil moisture geophysical data, version 4. NASA National Snow and Ice Data Center Distributed Active Archive Center 10.5067/KPJNN2GI1DQR Accessed: June 9, 2019.

[R107] Reichle R, Gabrielle J, De Lannoy M, Liu Q, Ardizzone J, Chakraborty P, (2017). Assessment of the SMAP Level-4 surface and root-zone soil moisture product using in situ measurements. Journal of Hydrometeorology, 18(10), 2621–2645. 10.1175/JHM-D-17-0063.1 PMC6196324 30364509

[R108] Robertson DM, & Saad DA (2014). SPARROW models used to understand nutrient sources in the Mississippi/Atchafalaya River Basin. Journal of Environmental Quality, 42(5), 1422–1440. 10.2134/jeq2013.02.0066 24216420

[R109] Ruddell BL, Drewry DT, & Nearing GS (2019). Information theory for model diagnostics: structural error is indicated by trade-off between functional and predictive performance. Water Resources Research, 55(8), 6534–6554. 10.1029/2018WR023692

[R110] Salas FR, Somos-Valenzuela MA, Dugger A, Maidment DR, Gochis DJ, David CH, (2018). Towards real-time continental scale streamflow simulation in continuous and discrete space. JAWRA Journal of the American Water Resources Association, 54(1), 7–27. 10.1111/1752-1688.12586

[R111] Schewe J, Heinke J, Gerten D, Haddeland I, Arnell NW, Clark DB, (2014). Multimodel assessment of water scarcity under climate change. Proceedings of the National Academy of Sciences, 111(9), 3245–3250. 10.1073/pnas.1222460110 PMC3948304 24344289

[R112] Schnitkey G (2013). Concentration of corn and soybean production in the U.S. Department of Agricultural and Consumer Economics. University of Illinois at Urbana-Champaign Available online at: http://farmdocdaily.illinois.edu/2013/07/concentration-corn-soybean-production.html Accessed: May 3, 2019).

[R113] Schuol J, Abbaspour KC, Yang H, Srinivasan R, & Zehnder AJB (2008). Modeling blue and green water availability in Africa. Water Resources Research, 44(7). 10.1029/2007WR006609

[R114] Secchi S, Gassman PW, Jha M, Kurkalova L, & Kling CL (2011). Potential water quality changes due to corn expansion in the Upper Mississippi River Basin. Ecological Applications, 21(4), 1068–1084. 10.1890/09-0619.1 21774414

[R115] Shen C, & Phanikumar MS (2010). A process-based, distributed hydrologic model based on a large-scale method for surface-subsurface coupling. Advances in Water Resources, 33(12), 1524–1541. 10.1016/j.advwatres.2010.09.002

[R116] Shook KR, & Pomeroy JW (2011). Memory effects of depressional storage in northern Prairie hydrology. Hydrological Processes, 25(25), 3890–3898. 10.1002/hyp.8381

[R117] Srinivasan R, Zhang X, & Arnold JG (2010). SWAT ungauged: Hydrological budget and crop yield predictions in the Upper Mississippi River Basin. Transactions of the ASABE, 53(5), 1533–1546. 10.13031/2013.34903

[R118] Tao B, Tian H, Ren W, Yang J, Yang Q, He R, (2014). Increasing Mississippi river discharge throughout the 21st century influenced by changes in climate, land use, and atmospheric CO_2_. Geophysical Research Letters, 41, 4978–4986. 10.1002/2014GL060361

[R119] Thornton PE, Thornton MM, Mayer BW, Wei Y, Devarakonda R, Vose RS, & Cook RB (2018). Daymet: Daily surface weather data on a 1-km grid for North America, version 3. Oak Ridge, Tennessee, USA: ORNL DAAC 10.3334/ORNLDAAC/1328

[R120] Thorslund J, Jarsjo J, Jaramillo F, Jawitz JW, Manzoni S, Basu NB, (2017). Wetlands as large-scale nature-based solutions: Status and challenges for research, engineering and management. Ecological Engineering, 108, 489–497. 10.1016/j.ecoleng.2017.07.012

[R121] Tootchi A, Jost A, & Ducharne A (2019). Multi-source global wetland maps combining surface water imagery and groundwater constraints. Earth System Science Data, 11(1), 189–220. 10.5194/essd-11-189-2019

[R122] Tullos D (2018). Opinion: How to achieve better flood-risk governance in the United States. Proceedings of the National Academy of Sciences, 115(15), 3731–3734. 10.1073/pnas.1722412115 PMC5899478 29636426

[R123] Turner RE, Rabalais NN, & Justic D (2008). Gulf of Mexico hypoxia: Alternate states and a legacy. Environmental Science and Technology, 42(7), 2323–2327. 10.1021/es071617k 18504960

[R124] USACE (2018). National Inventory of Dams. U.S. Army Corps of Engineers https://nid.sec.usace.army.mil, Accessed: April 3, 2019.

[R125] USEPA (2015). Connectivity of streams and wetlands to downstream waters: A review and synthesis of the scientific evidence (final report). U.S. Environmental Protection Agency, Washington, DC, EPA/600/R-14/475F.

[R126] USGS (2018a). National elevation dataset: U.S. Geological Survey National Map Viewer. http://viewer.nationalmap.gov/viewer/

[R127] USGS (2018b). National Water Information System: U.S. Geological Survey Water Data for the Nation https://waterdata.usgs.gov/nwis, Accessed: September 10, 2018.

[R128] Vanderhoof MK, Alexander LC, & Todd MJ (2016). Temporal and spatial patterns of wetland extent influence variability of surface water connectivity in the Prairie Pothole Region, United States. Landscape Ecology, 31(4), 805–824. 10.1007/s10980-015-0290-5

[R129] Veldkamp TIE, Wada Y, Aerts JCJH, Döll P, Gosling SN, Liu J, (2017). Water scarcity hotspots travel downstream due to human interventions in the 20th and 21st century. Nature Communications, 8(1), 15,697 10.1038/ncomms15697 PMC5481728 28643784

[R130] Voldseth RA, Johnson WC, Gilmanov T, Guntenspergen GR,& Millett BV (2007). Model estimation ofland-use effects on water levels of northern Prairie wetlands. Ecological Applications, 17(2), 527–540. 10.1890/05-1195 17489257

[R131] Wang X, Yang W, & Melesse AM (2008). Using hydrologic equivament weltand concept with in SWAT to estimate streamflow in watersheds with numerous wetlands. Transactions of the ASABE, 51(1), 55–72. 10.13031/2013.24227

[R132] Wilcox BP, Dean DD, Jacob JS, & Sipocz A (2011). Evidence of surface connectivity for Texas Gulf Coast Depressional wetlands. Wetlands, 31(3), 451–458. 10.1007/s13157-011-0163-x

[R133] Wing OEJ, Bates PD, Smith AM, Sampson CC, Johnson KA, Fargione J, & Morefield P (2018). Estimates of present and future flood risk in the conterminous United States. Environmental Research Letters, 13(3), 034023 10.1088/1748-9326/aaac65 PMC11977400 40201223

[R134] Wise EK, Woodhouse CA, McCabe GJ, Pederson GT, & St-Jacques J-M (2017). Hydroclimatology of the Missouri River Basin. Journal of Hydrometeorology, 19(1), 161–182. 10.1175/jhm-d-17-0155.1

[R135] Wood EF, Roundy JK, Troy TJ, van Beek LPH, Bierkens MFP, Blyth E, (2011). Hyperresolution global land surface modeling: Meeting a grand challenge for monitoring Earth’s terrestrial water. Water Resources Research, 47(5). 10.1029/2010WR010090

[R136] Wu Q, & Lane CR (2016). Delineation and quantification of wetland depressions in the Prairie Pothole Region ofNorth Dakota. Wetlands, 36(2), 215–227. 10.1007/s13157-015-0731-6

[R137] Wu Q, Lane CR, Wang L, Vanderhoof MK, Christensen JR, & Liu H (2019). Efficient delineation of nested depression hierarchy in digital elevation models for hydrological analysis using level-set method. Journal of the American Water Resources Association, 55(2), 354–368. 10.1111/1752-1688.12689 PMC7995241 33776405

[R138] Wu Y, Liu S, & Abdul-Aziz OI (2012). Hydrological effects of the increased CO_2_ and climate change in the Upper Mississippi River Basin using a modified SWAT. Climatic Change, 110(3–4), 977–1003. 10.1007/s10584-011-0087-8

[R139] Yaeger MA, Housh M, Cai X,& Sivapalan M (2014). An integrated modeling framework for exploring flow regime and water quality changes with increasing biofuel crop production in the U.S. Corn Belt. Water Resources Research, 50(12), 9385–9404. 10.1002/2014WR015700

[R140] Yamazaki D, Trigg MA, & Ikeshima D (2015). Development of a global ~90m water body map using multi-temporal Landsat images. Remote Sensing of Environment, 171, 337–351. 10.1016/j.rse.2015.10.014

[R141] Yang L, Jin S, Danielson P, Homer C, Gass L, Bender SM, (2018). A new generation of the United States National Land Cover Database: Requirements, research priorities, design, and implementation strategies. ISPRS Journal of Photogrammetry and Remote Sensing, 146, 108–123. 10.1016/j.isprsjprs.2018.09.006

[R142] Yen H, White MJ, Arnold JG, Keitzer SC, Johnson MVV, Atwood JD, (2016). Western Lake Erie Basin: Soft-data-constrained, NHDPlus resolution watershed modeling and exploration of applicable conservation scenarios. Science of the Total Environment, 569-570,1265–1281. 10.1016/j.scitotenv.2016.06.202 27387796

[R143] Yu F, & Harbor JM (2019). The effects of topographic depressions on multiscale overland flow connectivity: A high-resolution spatio-temporal pattern analysis approach based on connectivity statistics. Hydrological Processes, 33(10), 1403–1419. 10.1002/hyp.13409

[R144] Zalewski M, Arduino G, Bidoglio G, Junk W, Cullmann J, Uhlenbrook S, (2018). Low cost, nature-based solutions for managing aquatic resources: Integrating the principles of ecohydrology and the circular economy. Ecohydrology and Hydrobiology, 18(4), 309–310. 10.1016/j.ecohyd.2018.12.001

[R145] Zdankus N, Vaikasas S, & Sabas G (2008). Impact of a hydropower plant on the downstream reach of a river. Journal of Environmental Engineering and Landscape Management, 16(3), 128–134. 10.3846/1648-6897.2008.16.128-134

[R146] Zhou G, Wei X, Chen X, Zhou P, Liu X, Xiao Y, (2015). Global pattern for the effect of climate and land cover on water yield. Nature Communications, 6(1), 5918 10.1038/ncomms6918 25574930

[R147] Zhou T, Nijssen B, Gao H, & Lettenmaier DP (2016). The contribution of reservoirs to global land surface water storage variations. Journal of Hydrometeorology, 17(1), 309–325. 10.1175/JHM-D-15-0002.1

