# Supplementary material for: Surface Depression and Wetland Water Storage Improves Major River Basin Hydrologic Predictions
Source: Water Resour Res. Author manuscript; Available in PMC 2021 Jul 6. (PMC7751708; doi:10.1029/2019WR026561)
Supplement: Supplement1 [file NIHMS1611593-supplement-Supplement1.pdf]

AGU PUBLICATIONS

Water Resources Research

Supporting Information for

Surface depression and wetland water storage improves major river basin hydrologic predictions

Adnan Rajib[1], Heather E. Golden[2], Charles R. Lane[2], and Qiusheng Wu[3]

[1]Department of Environmental Engineering, Texas A&M University, Kingsville, Texas, USA; previously at the Oak Ridge Institute for Science and Education, US Environmental Protection Agency, Office of Research and Development, Cincinnati, Ohio, USA.

[2]US Environmental Protection Agency, Office of Research and Development, Cincinnati, Ohio, USA.

[3]Department of Geography, University of Tennessee, Knoxville, Tennessee, USA.

Contents of this file

Figures S1 to S5
Tables S1

Introduction

Figures S1-S3 summarize supplementary GIS data to allow efficient reproducibility of the Upper Mississippi River Basin (UMRB) model. Figures S4-S5 offer additional insights on the findings presented in this paper. Table 1 lists the parameters for model calibration.

a. Extent of subsurface (tile) drainage in the UMRB model

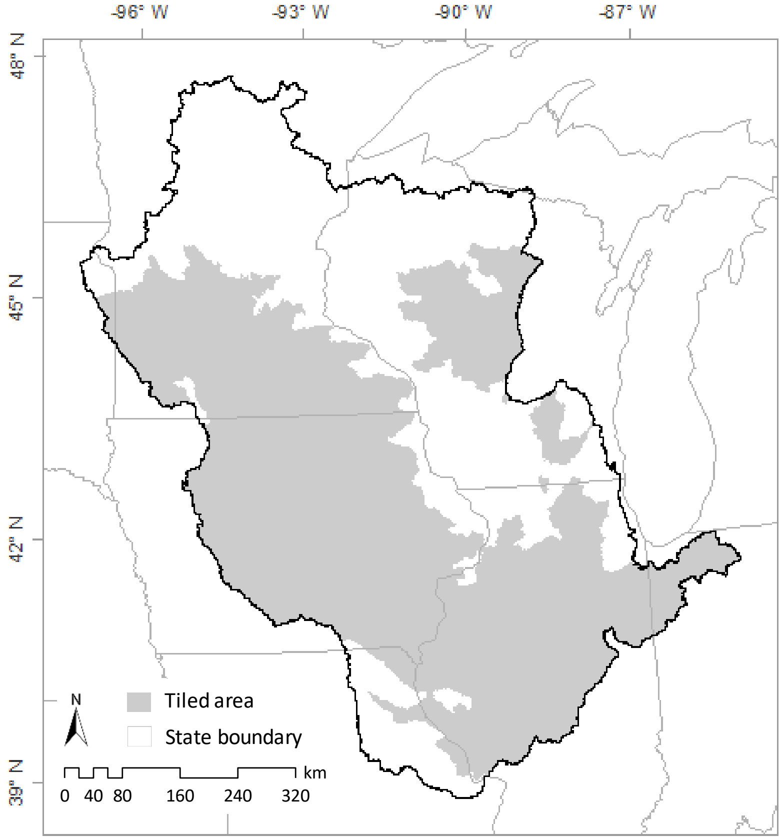

b. Tile drainage parameter values used in the UMRB model

Parameter	Definition*	Value**
DDRAIN	Depth to tile drain	1200 mm
DEP_IMP	Depth to impervious layer	1300 mm
TDRAIN	Time to drain the soil to field capacity	36 hr
GDRAIN	Time for tile flow to reach a stream (lag)	72 hr

* see Neitsch et al. (2011)

**Moriasi et al. (2012); Hutchinson and Christiansen (2013)

Figure S1. Inclusion of tile drainage in model simulations. See section 2.1 for details.

a. Reservoirs in the UMRB model

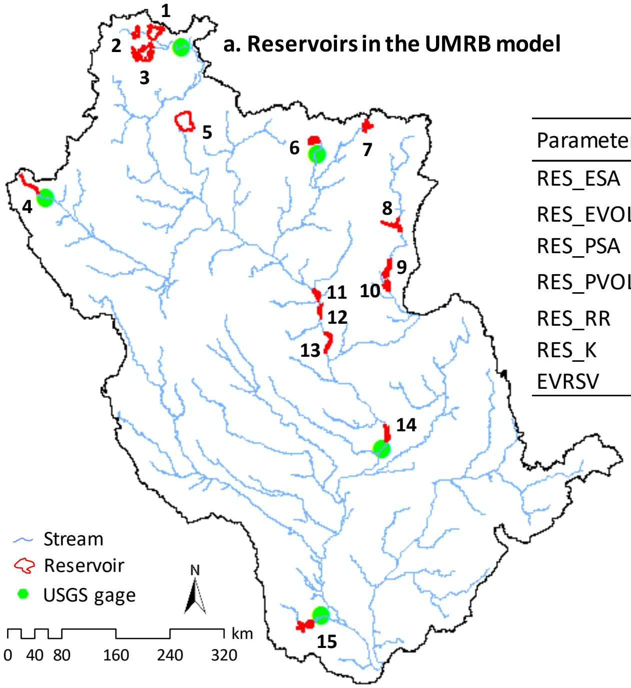

b. Parameter definition

Parameter	Unit	Definition (Neitsch et al., 2011)
RES_ESA	ha	Maximum surface area
RES_EVOL	$x10^4$ m^3	Maximum volume (storage capacity)
RES_PSA	ha	Surface area at normal operating condition
RES_PVOL	$x10^4$ m^3	Water volume at normal operating condition
RES_RR	m^3/s	Spillway design release rate
RES_K	mm/hr	Hydraulic conductivity
EVRSV	dimensionless	Lake Evaporation Rate

c. Reservoir hydraulics and storage-discharge parameter values (source: National Inventory of Dams)

Location	RES_ESA	RES_EVOL	RES_PSA	RES_PVOL	RES_RR	RES_K	EVRSV	Daily outflow data**
1, 2 & 3*	134785	151718	75069	87577		0.20	0.60	USGS #05211000
4	9946	25286	4894	12443		0.20	0.60	USGS #05292000
5	259458	82026	51892	16405	1000	0.20	0.60	
6	9428	41322	6192	27137		0.20	0.60	USGS #05356000
7	9886	29480	5584	16652	453	0.20	0.60	
8	3176	15887	3157	15789	4248	0.20	0.60	
9	10044	65374	9381	61057	8184	0.20	0.60	
10	8665	21586	6734	16775	9231	0.20	0.60	
11	11898	12952	5439	5921	7730	0.20	0.60	
12	20804	32070	8418	12976	7759	0.20	0.60	
13	28120	57974	11787	24300	7872	0.20	0.60	
14	14163	23683	11787	19657		0.20	0.60	USGS #05420500
15	8539	229674	2388	64222		0.20	0.60	USGS #05507800

* Considered as a single reservoir; the area and volume listed here are aggregated values

** Gage station located at the dam/lock or at a proximal downstream location; data accessible from https://waterdata.usgs.gov/nwis

Figure S2. Inclusion of major lakes and reservoirs in model simulation. See section 2.2 for details.

Streamflow gage stations for model calibration-verification

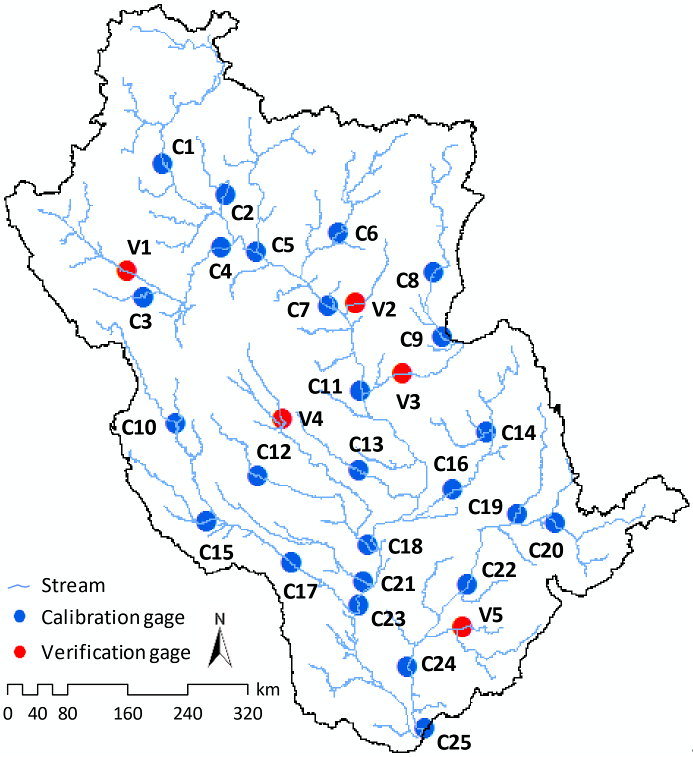

	Map location	USGS gage ID	KGE value	
			Excluding depressions	*Including* depressions
Calibration	C1	05267000	0.70	0.64
	C2	05286000	0.82	0.76
	C3	05317000	0.78	0.79
	C4	05330920	0.72	0.81
	C5	05344500	0.83	0.81
	C6	05365500	0.89	0.88
	C7	05378500	0.85	0.79
	C8	05400760	0.83	0.84
	C9	05404000	0.71	0.72
	C10	05476750	0.67	0.67
	C11	05389500	0.84	0.76
	C12	05451500	0.79	0.74
	C13	05421740	0.63	0.60
	C14	05437050	0.55	0.55
	C15	05484650	0.76	0.79
	C16	05443500	0.64	0.68
	C17	05489500	0.76	0.79
	C18	05465500	0.80	0.88
	C19	05552500	0.74	0.73
	C20	05527500	0.79	0.86
	C21	05474000	0.77	0.81
	C22	05568500	0.67	0.70
	C23	05474500	0.84	0.84
	C24	05586100	0.81	0.82
	C25	05587450	0.86	0.86
Verification	V1	05316580	0.65	0.78
	V2	05382000	0.84	0.85
	V3	05407000	0.67	0.69
	V4	05458500	0.90	0.85
	V5	05582000	0.77	0.78

Figure S3. Gage station ID and corresponding streamflow simulation performance (in terms of Kling-Gupta Efficiency, KGE) at each of the calibration-verification sites. The gage IDs can be used to access streamflow data from https://waterdata.usgs.gov/nwis.

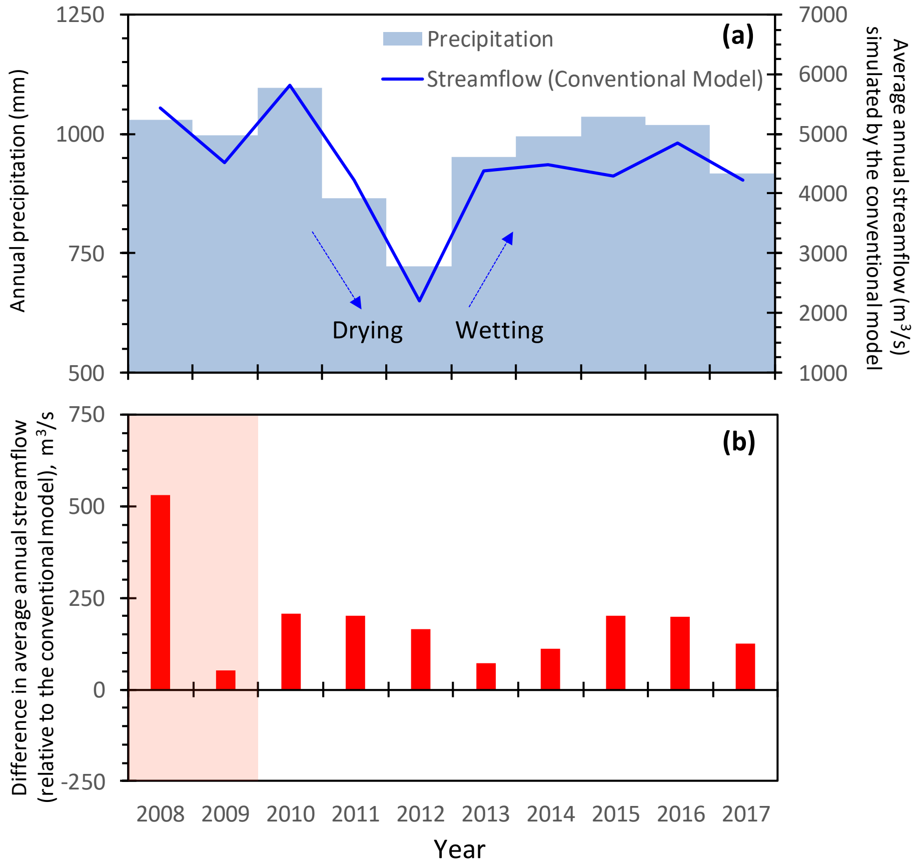

Figure S4. (a) Temporal variability of basin-average annual precipitation (mm) and average annual streamflow (m3/s) at the basin outlet simulated by the conventional "no-depression" model. A sequential drying and wetting phase across the 10-year simulation period is apparent both from the precipitation and streamflow data (schematically marked with arrows); (b) Difference in simulated streamflow (= conventional model output – depression-integrated model output). The highlighted years in (b) shows that surface depression storage may have minimal effect on the basin's overall hydrologic response between two successive "extremely" wet years (e.g., historic flooding of 2008 and similarly wet conditions in 2009; Holmes et al., 2010). However, if drying occurs following a wet condition (e.g., 2010-2012), effects of surface depression storage again become notable in the model.

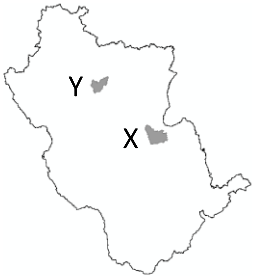

	X	Y
Precipitation	930 mm/year	870 mm/year
Depression volume	88 x 10^6 m^3 *	522 x 10^6 m^3 **
Land use	45% forest	40% agriculture
Soil	Moderately drained	Poorly drained
Slope	8%	1-4%

* mostly forested wetlands; ** non-forested wetlands

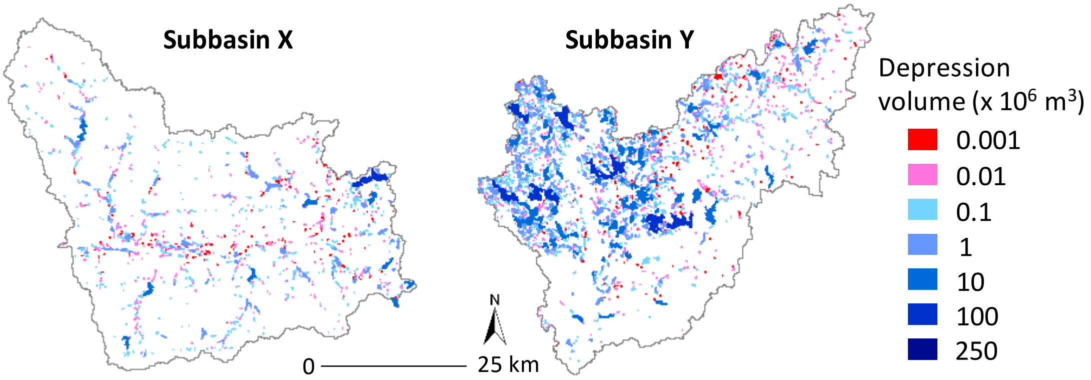

Figure S5. Climatic and geophysical characteristics of two similarly-sized subbasins which caused identical hydrologic response despite largely different surface depression storage capacities. See section 3.2 for details.

No.	Parameter [a]	Definition [b]	Spatial scale	Initial range
1	ALPHA_BF	Baseflow recession constant (days)	HRU	0.001 – 1
2	CH_K2	Channel hydraulic conductivity (mm/hr)	Subbasin	5 – 100
3	CH_N2	Main channel Manning's *n*	Subbasin	0.001 – 0.15
4	CN2	Curve number (moisture condition II)	HRU	-0.25 – 0.25
5	SURLAG	Surface runoff lag coefficient (days)	Basin	0.05 – 24
6	EPCO	Plant uptake compensation factor	HRU	0.01 – 1
7	ESCO	Soil evaporation compensation factor	HRU	0.01 – 1
8	GW_DELAY	Groundwater delay (days)	HRU	-10 – 10
9	GW_REVAP	Groundwater "revap" coefficient	HRU	0.01 – 0.2
10	GWQMN	Threshold depth for return flow (mm H_2O)	HRU	0.01 – 5000
11	REVAPMN	Re-evaporation threshold (mm H_2O)	HRU	0.01 – 500
12	TIMP	Snow pack temperature lag factor	Basin	0 – 1
13	SFTMP	Snowfall temperature (^oC)	Basin	-1.5 – 1
14	SMFMN	Min snowmelt factor (mm H_2O/^oC-day)	Basin	0 – 10
15	SMFMX	Max snowmelt factor (mm H_2O/^oC-day)	Basin	0 – 10
16	SMTMP	Snowmelt base temperature (^oC)	Basin	0 – 3
17	WET_K	Wetland hydraulic conductivity (mm/hr)	Subbasin	0.2 [c]
18	WETEVcoef	Wetland evaporation coefficient	Subbasin	0.6 [c]

[a] Each parameter, except CN2 and GW_DELAY, was iterated such that the original value was *replaced* by a value from respective initial ranges. During every iteration, a value from GW_DELAY's initial range was *added* to its original value; original value of CN2 was *multiplied* by an adjustment factor (1+ a value from the initial range).

[b] See Neitsch et al. (2011) for detail description of these parameters.

[c] Parameters related to surface depression storage, included only in the *depression-integrated* configuration. These parameters were kept consistent throughout the basin. Suitable values for these parameters were adopted from Golden et al. (2019).

Table S1. Parameters included in the calibration of the UMRB model.
